# Conceptualizing *in situ* energy station for Mars exploration

**DOI:** 10.1093/nsr/nwag043

**Published:** 2026-01-27

**Authors:** Yuzhuo Yang, Peng Tan, Hua Tian, Lingfeng Shi, Gequn Shu

**Affiliations:** Department of Thermal Science and Energy Engineering, University of Science and Technology of China, China; Deep Space Exploration Laboratory, China; Department of Thermal Science and Energy Engineering, University of Science and Technology of China, China; Department of Thermal Science and Energy Engineering, University of Science and Technology of China, China; Department of Thermal Science and Energy Engineering, University of Science and Technology of China, China; Deep Space Exploration Laboratory, China; Department of Thermal Science and Energy Engineering, University of Science and Technology of China, China

## Abstract

This perspective underscores Martian air as the working medium for the space nuclear system integrated with heat-to-electricity and chemical conversion, paving the way for multimodal resource transformation on Mars.

Recently, China has announced plans to launch the Tianwen-3 Mars sample return mission in 2028, searching for potential biosignatures [1]. The USA has also announced its sampling return and landing draft plans for the next 20 years [2]. With the development of Space exploration technology, it is not too far-fetched to presume that one day humans will land on Mars. A significant challenge is how to address the energy source once humans land on Mars. The latest report from the National Aeronautics and Space Administration (NASA) highlights that future Mars bases will need hundreds of kilowatts for surface power [3]. Human life on Mars also requires various types of resources, such as oxygen, fuel and water. Due to the vast distance, relying on bringing everything above needed from Earth is enormously expensive [4].

*In situ* resource utilization (ISRU) is defined as the conversion of local resources to promote sustainable and cost-effective space exploration. The gear ratio for delivery of cargo to the Martian surface from low Earth orbit is about 8–10 (lunar is about 2.5), and the required liquid-oxygen mass for ascent from Mars is 3.7–5 times greater than ascent from the Moon [5]. It follows that Mars ISRU has greater potential in terms of reducing spacecraft payload. *Science* has listed ‘*How can we develop manufacturing systems on Mars?*’ as one of the 125 cutting-edge scientific questions, highlighting that addressing energy issues through Mars ISRU is a breakthrough solution [6].

In comparison to Earth, the Martian atmosphere has lower pressure (∼600 Pa) and temperature (∼210 K). It is primarily composed of carbon dioxide (∼96%), with nitrogen (∼2%) and argon (∼2%) as secondary components [7]. These gases can serve as a heat transfer medium and are *in situ* sources of carbon and oxygen elements. Effectively harnessing the thin atmosphere to support diverse resource demands is a significant challenge. We believe that Martian air as the working medium for the space nuclear system, integrated with heat-to-electricity and chemical conversion, is paving the way for multimodal resource transformation on Mars (Fig. 1). A more comprehensive research background is provided in the Supplementary data. Recently, the individual Mars ISRU technologies have been rapidly developing, using the same working medium and relying on temperature and pressure adjustments. Space energy systems aim for high compactness. These shared needs lay the foundation for efficient matching (energy and material flows) and multimodal resource transformation. The implementation pathway includes three main steps: (i) Martian atmospheric capture; (ii) *in situ* power generation and storage; and (iii) life-support resources transformation, which may afford a fruitful insight.

**Figure 1.**
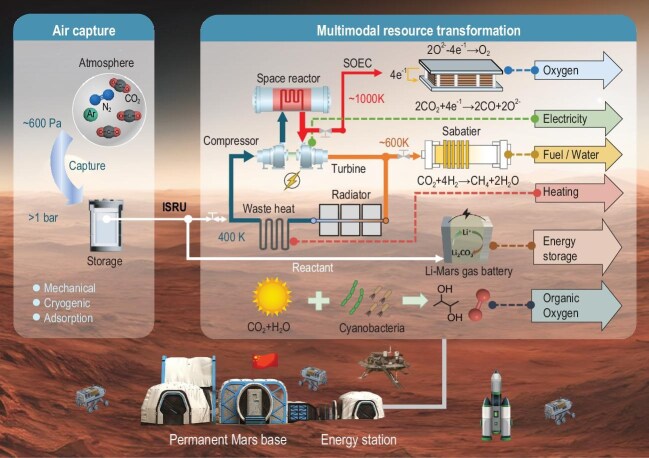
Potential technological pathways for ISRU of the Martian atmosphere. Left panel: collecting and processing the Martian atmosphere to a working state. Achieved through mechanical compression, cryogenic trapping or temperature swing adsorption. Middle panel: utilizing the Martian air as a working medium for heat-to-electricity conversion (nuclear reactor) and energy storage. Right panel: coupled chemical conversion module and biotechnology satisfy the fundamental resource needs for human survival on Mars.

***Martian atmospheric capture.*** Collecting and compressing Martian air from atmospheric pressure (600–800 Pa) to working pressure (>100 kPa) is fundamental for developing *in situ* energy conversion. Potential methods include mechanical compression, cryogenic trapping or adsorption. NASA’s Perseverance rover has proved the reliability of mechanical compression on Mars (reaching 80 kPa). The adiabatic efficiency and peak pressure ratio of NASA’s prototype (the first stage) are approximately 35% and 17.9, respectively, with an optimized design expected to exceed 74% [8]. After an initial pressure boost, the atmosphere continues to undergo 2–3 compression stages (single-pressure ratio ∼ 5) to reach the target pressure. Similar miniature stages-in-series compressors have been developed and tested on the ground [9]. The rotating parts may encounter lubrication issues in low-gravity conditions, where gas is difficult to separate from the lubricating oil. Mechanical compression may be suitable only for small-scale, short-term testing on the Perseverance rover, because current designs and testing data do not demonstrate its long-life performance. Cryogenic trapping is currently in the testing phase. It can produce pure CO_2_ (∼99%) with a single stage (pressure ratio exceeding 10 000), and has high reliability without moving components. Its drawback is high power consumption (2–3 times higher than mechanical compression, >0.87 Wh/g), mainly due to the low efficiency (10%–15%) of the space cryocooler operating in the Mars CO_2_ deposition temperature (∼148 K) [10]. Based on the affinity of solid adsorbent materials for the CO_2_ molecule (such as zeolite 13X, Mg-MOF-74), a temperature swing adsorption cycle can be established between the low-temperature environment and the heat source. When low-grade waste heat is available, thermally driven adsorption compression becomes more attractive, as it does not require electricity and has no moving parts. However, initial tests have revealed challenges such as low thermal conductivity and limited rates [11]. Overall, the choice between the above methods requires careful consideration of the tolerance of impurity components (N_2_ and Ar), designed collection pressure, flow rate, weight, service period and power consumption.

***In situ power generation and storage.*** Given the sandstorm climate, the reliance on solar energy may need to be reconsidered for long-term human-crewed missions [3]. Scientists aim to use high-energy-density, environmentally adaptive nuclear reactors to power human settlements on Mars [12,13]. Heat-to-electricity conversion based on the thermodynamic cycle (Brayton or Stirling) has provided several tens of kilowatts during the night and dust storms [13]. Recent studies have shown that Martian air, with excellent thermal stability, high specific heat capacity and large molecular weight, is an ideal working medium for power generation [14]. Its theoretical performance may surpass the widely studied helium–xenon mixture, with efficiency and power density expected to reach 28% and 45 W/kg, respectively. Small amounts of leaked gas can be replenished by the atmospheric capture device, mitigating the potential risk of high-pressure medium leakage within moving components under extreme environments. Martian air is suitable for operating in a closed subcritical pattern, which avoids safety issues from high-pressure operations and phase changes in microgravity [15], while reducing power consumption at the gas collection end. However, the changing Mars environment (such as sky temperature, solar flux and local wind speed) may cause the actual operating parameters within the thermodynamic cycle to deviate from the designed state, leading to real-time output power fluctuations (within 10%) [16]. Additionally, wind turbines (utilizing atmospheric kinetic energy) can generate several kilowatts of electricity during a Martian dust storm (lasting for several weeks) as a back-up energy source for heat-to-electricity conversion [17]. Lithium–Mars gas batteries provide an *in situ* electricity storage solution to balance the above-mentioned output power fluctuations, including the uncertainty of wind energy. A trace amount of oxygen within the Martian atmosphere can enhance the lithium–Mars gas battery’s performance, providing high energy density (765 Wh/kg) and long cycling lifespan (1350 h) in the low-temperature environment (273 K), potentially replacing lithium-ion batteries (160–350 Wh/kg) [18]. Noticeably, lithium–Mars gas batteries are expected to degrade or even significantly fail at Martian near-surface pressure. When the operational pressure increases from 6 to 600 kPa, the specific capacity significantly increases from 665 to 11 546 mAh/g [19]. Additionally, adapting temperature can shift the reaction pathway and improve discharge capacity [20]. Therefore, using an atmospheric capture device to provide appropriate reaction conditions is indispensable. Whether for power generation or energy storage, Martian air can play a key role as an energy carrier.

***Life-support resources transformation.*** Combining Martian air heat-to-electricity conversion with advanced CO_2_ chemical engineering will provide essential life-support resources (heating, fuel and oxygen) for human settlements, significantly reducing the rocket’s mission costs. NASA’s Mars habitat (accommodating four astronauts) theoretically requires 3300 W of continuous heating capacity to prevent internal freezing [21]. The cold-end temperature of heat-to-electricity conversion is relatively low (∼400 K), and sufficient waste heat is available. Recovering part of low-temperature waste heat can enable combined power and heating production. Based on the Sabatier process, producing fuel (CH_4_) and water by reacting CO_2_ from Martian air with hydrogen at moderate conditions (423–623 K, 100–300 kPa) is a promising approach [22]. Studies show that Mars has abundant groundwater/ice [23], and hydrogen can be produced via water electrolysis to overcome sourcing and transportation challenges. Conducting heat to the ice regolith to sublimate or evaporate (thermal mining) [15] and deploying excavation robots (such as NASA’s RASSOR) to extract and dry hydrated minerals [24] are both potential technologies for extracting groundwater/ice. The temperature and pressure conditions of Martian air after expansion from the turbine are within the ideal range for the Sabatier reaction. Extracting a portion of the exhaust gases can both provide feedstock (CO_2_) for the Sabatier reaction and enable thermal cascade utilization. Given the uneven distribution of groundwater/ice [25], it is crucial to assess the soil conditions of landing sites before deploying Sabatier technology. Approximately 75%–80% of the propellant mass of the Mars Ascent Vehicle (methane–oxygen engine) is liquid oxygen [26]. The currently feasible *in situ* oxygen production technology involves electrically heating collected Martian air to 1073 K, followed by electrolyzing CO_2_ to produce oxygen using a solid oxide electrolysis cell (SOEC) with a scandia-stabilized zirconia electrolyte. This technology was demonstrated by the Perseverance rover on Mars, which converts 30%–50% of the CO_2_ to carbon monoxide and oxygen (50 g, as of 2021) [27]. NASA is working to expand this technology for larger-scale oxygen production (14 months, 30 tons) [12]. Recent studies have proposed a monolithic gyroid SOEC stack, removing seals and connectors, which could reduce weight by an order of magnitude compared to NASA’s planar stack, further expanding the application potential [28]. Integrating the SOEC module with heat-to-electricity conversion can enable combined power and oxygen production. Both systems’ operating temperature and pressure are matched. The power conversion module can easily provide stable and suitable reactants for the SOEC module, eliminating the need for atmospheric pre-compression and heat transfer components before the SOEC module. Notably, on Earth, coupling heat-to-electricity conversion with chemical electrolysis module has already proved to be feasible and efficient theoretically and experimentally [29]. Furthermore, some microbes demonstrate strong resilience in the Martian environment, making them ideal tools for ISRU [30]. For example, cyanobacteria convert the atmosphere carbon dioxide into oxygen and glucose via photosynthesis, which is then converted into a specific fuel (2,3-butanediol, 2,3-BDO) by engineered microbes [31]. Integrating biotechnology could reduce energy demands in intensive ISRU production.

***Assessment of technical potential.*** We analyze the energy processes within the proposed design framework for atmosphere ISRU, highlighting its thermodynamic advantages (Figs S1 and S2). To clarify the technical potential of the proposed design framework, we reference NASA’s early crewed Mars mission energy requirements (Table S1) and have developed a preliminary model to assess the flow rates, power flows and component weights at key points for the design framework (Supplementary data). The proposed ISRU framework (42 861 W and 6313 kg) for liquid-oxygen production (30 804 kg in total) can reduce the propellant transportation mass by 60% (Figs S3 and S5). Compared to solar arrays and Stirling engines, *in situ* power generation demonstrates advantages in terms of mass and integration modularization (Fig. S4 and Table S2). We conceptualize an *in situ* energy station to demonstrate its feasibility in solving both propellant production and energy requirements of the Mars habitat (Fig. S6 and Table S3). Compared to the energy station without ISRU (Table S4), the payload transported by the spacecraft to the Martian surface will be reduced by dozens of tons. Overall, the concept of an integrated energy system based on atmosphere ISRU is engineering-feasible. It can be considered as an independent facility that needs to be deployed before human arrival.

The Martian atmosphere, as a central medium for power generation, can integrate independent chemical conversions to realize a power-to-X function. This perspective synthesizes the common characteristic of independent Mars CO_2_ ISRU, and outlines a vision for the future pathway. The first crewed Mars mission is expected to materialize in the coming decades. However, related ISRU technologies are still in the conceptual experimentation and analysis phase. To narrow the gap for practical applications, the following directions may be considered in the future.

- Unveiling the Martian atmosphere properties: The CO_2_ concentration in the Martian atmosphere exhibits periodic fluctuations, dropping to as low as 78% during the southern hemisphere’s winter. Currently, there is no complete dataset on the experimental physical properties of the multi-component gas mixture. Only partial data for certain low-temperature ranges are available. The accuracy of the equation of state (GERG-2008 EOS) has only been experimentally validated in low-temperature ranges [14]. Conducting experiments with a simulated Martian atmosphere on the thermophysical property testing platform (viscosity, heat conductivity, specific heat capacity, temperature/pressure/density) is essential for future research.
- Key components development: Developing the compression-expansion-power generation integrated turbomachinery and high-temperature CO_2_ corrosion-resistant materials (>800°C, Inconel) is crucial for efficient energy conversion. Exploring suitable high-temperature sealing and insulating materials, electrode materials and electrolytes is necessary to enhance the efficiency and lifespan of chemical devices. For example, the electrolyte of lithium–Mars gas batteries could explore the transition from the current organic system to a solid-state system [32]. Artificial intelligence robots can also be used to identify *in situ* catalysts for chemical reactions [33]. Low density and low Reynolds number (∼700) increase the design complexity of Martian atmospheric capture. Currently, the isentropic efficiency of the prototype from the USA is only 35% compared to the theoretical boundary (60%–∼70%). An efficient, simplified inert gas removal device is crucial for cryogenic trapping and adsorption. Martian dust particles can obstruct radiator heat dissipation and clog gas collectors, necessitating the development of corresponding anti-dust coatings or piezoelectric actuators.
- Efficient integrated design: Space energy devices aim for high-energy-density and integrated design, while also facing the unique challenges posed by using Martian air as the working fluid. The unique environmental characteristics, such as low pressure (∼600 Pa) and low gravity (0.38 g), present fundamental differences compared to energy systems on Earth. Existing experience in CO_2_ heat transfer, transcritical CO_2_/supercritical CO_2_ (tCO_2_/sCO_2_) power cycle, and chemical reactions (pure CO_2_) may not be applicable. Additionally, the adsorption material characteristics under the Mars unique conditions are still not well understood. It is necessary to explore efficient matching paths for energy flow and material transfer within the complex energy conversion model, optimizing operational parameters, based on the resource demand objectives during the Earth–Mars transport window.
- Extreme environment control: The Martian energy system needs to have independent autonomous control capabilities. Diurnal temperature changes (±60 K), fluctuating atmospheric composition (78% to 96% CO_2_) and pressure variations (±200 Pa) result in unstable operating parameters (off-design conditions). For example, the cold-end temperature is highly sensitive to sky temperature, and extreme summer temperatures can cause thermal buildup within the reactor. A single control strategy is inadequate for adapting to Mars’ changing environment. A promising direction is to establish a dynamic model based on real climatic data, combined with the predictive capabilities of artificial intelligence [34], to obtain the components adjustment strategy under the changing Mars environment.

## Supplementary Material

nwag043_Supplemental_File
