# Supplementary material for: Conceptualizing in situ energy station for Mars exploration
Source: Natl Sci Rev. 2026 Jan 27;13(6):nwag043. doi: 10.1093/nsr/nwag043 (PMC13032873; doi:10.1093/nsr/nwag043)
Supplement: nwag043_Supplemental_File [file nwag043_supplemental_file.docx]

Supporting Material for

***Conceptualizing in-situ energy station for Mars exploration***

Yuzhuo Yang ^1,2^, Peng Tan ^1^, Hua Tian ^1^, Lingfeng Shi ^1,2,^*, Gequn Shu ^1,^*

**Author affiliations:**

*1. Department of Thermal Science and Energy Engineering, University of Science and Technology of China, Hefei, 230027, China*

*2. Deep Space Exploration Laboratory, Hefei 230026, China*

****Corresponding author:***

Gequn Shu, Email: [shugequn@ustc.edu.cn](mailto:shugequn@ustc.edu.cn).

Lingfeng Shi, Email: [slf@ustc.edu.cn](mailto:slf@ustc.edu.cn)

**This PDF file includes:**

1. A more comprehensive research background

2. Thermodynamic analysis for proposed design framework

3. Potential assessment for proposed design framework

4. Modeling method and MATLAB code

***A more comprehensive research background***

1. ***Energy source on Mars***

National Aeronautics and Space Administration (NASA) estimates that the power requirements for human Mars exploration will reach the scale of hundreds of kilowatts [1], with the highest energy consumption coming from ISRU production. Electricity is the foundation for supporting future Mars exploration missions. The most accessible energy sources on Mars are solar and wind energy, with photovoltaic technology being the predominant energy solution for current Mars rovers. NASA has attempted to develop large solar arrays (1000 m², with an average power output of 26~29 kW) to meet the electricity demands of future human exploration missions [2]. Due to factors such as low solar flux, dust storm damage, lack of day-night stability, and short lifespan on Mars (~1 year), NASA is compelled to reduce reliance on solar energy [1, 22]. Mars has an active atmospheric system [3], but the low atmospheric density limits the available wind energy resources for development. Research has found that in certain areas with abundant wind resources, wind turbines can generate power exceeding 24 kW for more than 35% of the time [4]. Wind energy could therefore be a more suitable supplementary power source for crewed exploration missions [4, 5]. Micro space nuclear reactor, with high energy density, long lifespan, and stable power output, have become a research focus for space-faring nations [6-8], especially for Mars and lunar bases with power demands of tens of kilowatts or more [9, 10]. The Perseverance rover, powered by a nuclear system, continues to operate normally since 2021.

1. ***Heat-to-electricity conversion on Mars***

Heat-to-electricity conversion is one of the key modules of space nuclear power system, directly impacting the overall output performance and mass. Thermoelectric materials, Stirling engines, and Brayton engines are the mainstream methods for space thermoelectric conversion. Thermoelectric materials have no moving parts, making them silent and noise-free, but their efficiency is relatively low (<10%) [11-13]. They are well-suited for small power sources (<1000W) or detectors, particularly in isotope nuclear power system [14]. Researchers are working on developing higher-efficiency multi-stage thermoelectric materials to support power outputs in the range of 1~10 kW [12, 15]. The application of low-efficiency thermoelectric materials in high-power scenarios poses challenges, as it can lead to significant increases in the mass of the reactor’s thermal power, shielding systems, and heat dissipation systems [16]. In contrast, high-efficiency dynamic thermoelectric conversion methods, such as Stirling and Brayton engines, are more competitive in high-power applications. Stirling engines offer excellent thermal efficiency. The working medium inside is high-pressure helium gas, which drives the reciprocating motion of the piston through pressure fluctuations within the cylinder, generating electricity [17]. Stirling engines involve isothermal expansion and compression during operation, which requires larger and more complex heat exchange systems. As a result, the single-unit power output and power density are relatively lower [18]. Compared to Brayton engines, Stirling engines have a technological advantage in the 1~10 kW power range [8, 19]. Recently, NASA has launched the Kilopower project, which focuses on developing a nuclear-powered Stirling system (1–10 kW) for future Mars missions [20]. The goal is to start with small-scale systems to gather valuable experience and data for the subsequent development of larger-scale nuclear power systems [20, 21]. The China Academy of Space Technology has pointed out that switching from Stirling engines to Brayton engines is the key to achieving the breakthrough of 50 kW power output [8]. NASA began research on space-based Brayton engines in the 1960s, with a particular focus on testing key components, especially the integrated compressor-turbine-generator unit. The output power ranged from 500 W to 30 kW. They also developed gas bearing systems to accommodate rotational speeds of up to 90,000 rpm [23]. The related technologies underwent rigorous reliability testing, with a total duration of approximately 50,000 hours [23]. In the early 2000s, NASA launched the Jupiter Icy Moons Orbiter (JIMO) project, with the preferred option being nuclear-powered Brayton engines in the 100~200 kW. A prototype of 30 kW engine was developed and tested at the Sandia National Laboratories [24]. NASA has evaluated the power requirements for manned Mars mission, estimating a need of approximately 50 kW. They pointed out that the space Brayton engine is the lightest solution [25]. In conclusion, the Space Brayton engine holds significant potential as a heat-to-electricity conversion method for future manned Mars missions [26]. NASA has since undertaken systematic development efforts aimed at adapting this technology [27].

1. ***Working fluid in Brayton engine on Mars***

The working fluid in a Space Brayton engine act as the energy transfer medium, directly determining the system's output performance and power density. Helium-xenon mixed gases are of significant interest as working fluids in NASA's space Brayton projects due to their excellent thermal conductivity, thermal stability, and compressibility [23]. China's latest space Brayton cycle proposal also selects helium-xenon rare gases as the working fluid [28]. Recent studies on helium-xenon Brayton engines have focused on gas properties [29], parameter design [30-32], variable operating conditions [33-36], transient control [37-40], rotating machinery [41], and heat transfer [42]. High density and compressibility properties of carbon dioxide (CO_2_) enable high-efficiency power generation and compact design, making it particularly suitable for space-constrained applications such as spacecraft and marine propulsion [43,44]. As early as the 1960s, researchers recognized the potential of CO_2_ as a working fluid for space Brayton engine. However, due to limitations in the development level of small-scale rotating machinery and compact heat exchanger at the time, this work could not be continued [45]. The required heat recovery capacity in the CO_2_ recuperator is nearly twice that of helium-xenon, but the large volume of the shell-and-tube heat exchanger is unacceptable. This issue has been alleviated with the emergence of high-strength, high-volume-density printed circuit heat exchangers [46,47]. Additionally, technological advancements have been made in the development of small-scale CO_2_ rotating machinery [48-50]. Recently, NASA developed a design for a megawatt-class Mars nuclear-powered spacecraft, comparing two working fluids: helium-xenon and supercritical CO_2_. The latter demonstrated promising thermal efficiency and power density, with plans for its application between 2030 and 2040 [51]. Mars has an atmosphere resource, primarily composed of CO_2_, followed by N_2_ and Ar. If Martian atmospheric resources are utilized effectively, it can address the potential leakage issues of dynamic power generation systems [49,50,52] and the challenges related to working fluid replenishment. This becomes particularly critical considering that the extraterrestrial environment may exacerbate leakage risks [53]. One approach is to directly transplant supercritical CO_2_ Brayton cycle technology for applications on Mars surface [54-56]. However, several engineering challenges arise. On the one hand, supercritical CO_2_ operates at very high pressures (~20 MPa), which poses safety concerns for crewed Mars missions [57] and places stringent requirements on atmospheric capture and purification. On the other hand, the power demand for initial crewed Mars exploration missions is relatively low (~50 kW), and at this scale, the volume flow rate of supercritical fluid within rotating machinery is small, making it difficult to achieve optimal isentropic efficiency [49,50]. Additionally, the thermal environment on Mars makes it challenging to cool the supercritical CO_2_ fluid to near its critical temperature (~305 K), preventing the advantage of its high efficiency from being realized [58,59]. We have unexpectedly discovered that the Martian atmosphere is nearly a perfect working medium and report unique work patterns on Mars [59]. The inertness, low adiabatic index, and large molecular characteristics of Martian air (CO_2_-Ar-N_2_ mixture) contribute to increased efficiency and power density. Compared to current mainstream rare gases, efficiency could improve by 7.4% to 20.0%, and power density could increase by 1.0% to 14.2%. Notably, substantial efficiency (>22%) can be achieved even at relatively low hot-end temperatures (<973K) [60]. In addition, compared to directly transplanting supercritical CO_2_ Brayton technology, operating in a subcritical mode with Martian air as working fluid can help mitigate safety risks, atmosphere capture issues, and the challenges of two-phase flow control (in microgravity). This approach could offer a safer and more efficient salutation for Brayton engine application on Mars. Recent research has revealed the potential of using Martian atmosphere as a working medium from both thermodynamic and system design perspectives [61]. It has also explored the feasibility of an open Brayton cycle configuration [62]. However, the open-cycle configuration may not be suitable for Mars' low atmospheric pressure environment. Factors such as low density and low Reynolds number make it difficult for the isentropic efficiency of the first-stage compressor to reach ideal condition (~80%). In fact, NASA's prototype demonstrated an isentropic efficiency of only 35% [63]. As an energy station for future Mars missions, considering the Martian air Brayton engine solely as an independent power generation unit has not fully explored its application potential. Mars habitats require not only electricity but also thermal energy and life-resources for human survival [87]. The Martian atmosphere contains abundant carbon and oxygen, as well as usable thermal energy from Brayton engine, making it possible to expand the system into an integrated energy station. Such a station could not only generate electricity but also support life-resource conversion, rocket propellant production, and thermal energy supply for Mars habitats.

1. ***In-situ resource utilization system on Mars***

Obtaining life resources and propellant is a key factor in enhancing the feasibility of crewed Mars exploration. Relying solely on Earth-based resupply is prohibitively expensive and unsustainable. NASA has conducted extensive research on converting Martian CO₂ and subsurface water ice into O₂ and CH₄ [64, 88]. Based on the Sabatier reaction and high-temperature solid oxide electrolysis (SOEC) principles, a series of test modules have been developed to advance the relevant electrochemical technologies. In a methane-liquid oxygen rocket system, oxygen accounts for up to 78% of the propellant mass. The simplicity and high benefit of CO₂ resource utilization technologies make them particularly suitable for early Mars missions [65, 66]. In NASA's Mars Mission Design Framework 5.0, it is pointed out that the first manned Mars mission will utilize Solid oxide electrolysis (SOE) technology to production oxygen, with methane being carried by the spacecraft itself. An In-Situ Resource Utilization (ISRU) plant, powered by nuclear system, will operate prior to human arrival [67]. In recent years, NASA has made significant progress in several key areas related to human Mars exploration, including the atmospheric resources capture [68-71], testing and integration of SOE stacks [72-74], oxygen production testing by the Perseverance rover [75, 76], and cryogenic oxygen storage [77-79]. These advancements are crucial for preparing for human missions to Mars. ISRU is an energy-intensive process that heavily relies on a stable and reliable power supply on Mars. NASA's Mars Oxygen In-Situ Resource Utilization Experiment (MOXIE) team designed a full-scale in-situ oxygen production device, considering factors like mass, volume, power, and risk. The team discovered that the peak power consumption for the system could reach nearly 30 kW [80, 81]. When combined with the power requirements for the Sabatier reactor, which can approach 80 kW [82]. Researchers have reported thermodynamic-level design results [83]. Currently, research on system-level integration of Mars ISRU remains insufficient, and the technical pathways are unclear. For example, the coupling and configuration of power systems are often addressed by simply listing different energy conversion methods [84, 85], while strategies and mechanisms for aligning energy and material flows within the system are still undefined.

Optimizing power usage and integrating these energy-intensive processes is a key challenge in making Mars missions more autonomous and sustainable. Developing a technically feasible, efficient, and highly integrated ISRU energy system design framework [86] holds significant scientific value for advancing sustainable manned Mars missions in the future.

1. **The contribution of this research**

The harsh environmental conditions and the vast distance for transportation present significant challenges to ensuring a stable resource supply for human activities on Mars. Utilizing in-situ Martian air as the working medium for energy transfer and material conversion, coupled with advanced thermodynamic cycles and chemical processes, could offer an innovative path to build sustainable energy station on Mars. This perspective highlights the key role of Martian atmosphere in multimodal resource conversion. We propose a design framework for future in-situ Martian energy systems, analyze the implementation pathways and current status of resource conversion, and demonstrate its potential to reduce rocket payload costs through thermodynamic analysis. Finally, we summarize the future challenges and directions in the field of multimodal resource conversion on Mars.

***Reference***

[1] National Aeronautics and Space Administration, Mars Surface Power Generation Challenges and Considerations. https://www.ntrs.nasa.gov/citations/20230015763 (2023), Accessed 31 Aug 2025.

[2] National Aeronautics and Space Administration, Mars Surface Solar Arrays: Part 2 (Power Performance). https://ntrs.nasa.gov/citations/20170006908 (2017), Accessed 31 Aug 2025.

[3] The Modern Near-Surface Martian Climate: A Review of In-situ Meteorological Data from Viking to Curiosity. Space Science Reviews, 2017, 212: 295-338.

[4] V L Hartwick, O B Toon, J K Lundquist, et al. Assessment of wind energy resource potential for future human missions to Mars. Nature Astronomy, 2023,7: 298-308.

[5] Vera Schorbach, Tilo Weiland. Wind as a back-up energy source for Mars missions. Acta Astronautica, 2022, 191: 472-478.

[6] Tien Nguyen. Powering Human Settlements in Space: Nuclear reactors might be the best electricity source for colonies on the moon and Mars. ACS Central Science, 2020, 6(4): 450-452.

[7] International Atomic Energy Agency, The Role of Nuclear Power and Nuclear Propulsion in the Peaceful Exploration of Space.

<https://www-pub.iaea.org/MTCD/Publications/PDF/Pub1197_web.pdf> (2005), Accessed 31 Aug 2025.

[8] Y Xia, J Li, R Zhai, et al. Application Prospect of Fission-Powered Spacecraft in Solar System Exploration Missions. Space: Science & Technology,2021,2021:15.

[9] Wu Weiren, Liu Jizhong, Zhao Xiaojin, et al. System engineering research and application foreground of space nuclear reactor power generators. Scientia Sinica Technologica, 2019, 49: 43477.

[10] National Aeronautics and Space Administration, Nuclear Systems Kilopower Overview. https://ntrs.nasa.gov/citations/20160012354 (2016), Accessed 31 Aug 2025.

[11] Dulyawich Palaporn, Sora-at Tanusilp, [Yifan Sun](https://www.zhizhen.com/s?sw=author%28Yifan+Sun%2C+%3Csup%3Ec%3C%2Fsup%3E%29), et al. Thermoelectric materials for space explorations. Materials Advances, 2024, 5: 5351-5364.

[12] Simiao Tang, Longxiang Zhu, Qiang Lian, et al. Design and optimization of three segmented thermoelectric generator for nuclear reactor application. Progress in Nuclear Energy, 2024, 173: 105243.

[13] Datas A, Marti A. Thermophotovoltaic energy in space applications: Review and future potential. Solar Energy Materials and Solar Cells, 2017, 161: 285-296.

[14] Bai Shengqiang, Liao Jingchen, Xia Xugui, et al. Research Progress of Thermoelectric Materials and Devices for Radioisotope Thermoelectric Generators. Journal of Deep Space Exploration, 2020, 7: 525-535.

[15] Simiao Tang, Chenglong Wang, Xiao Liu, et al. Experimental investigations on start‐up performance of static nuclear reactor thermal prototype. International Journal of Energy Research, 2020, 44: 3033-3048.

[16] Wu YiCan, Liu Chao, Jin Ming, et al. Design and R&D of megawatt lithium-cooled space nuclear reactor. Scientia Sinica Technologica, 2024, 54: 365-376.

[17] Yuhao Zhang, Yusheng Liao, Yuanshu Qu, et al. Dynamic thermal characteristics analysis of integrated space nuclear reactor with core and Stirling power conversion components. Energy, 2025, 315: 134458.

[18] Rui Yang, Junxiang Wang, Ercang Luo. Revisiting the evaporative Stirling engine: The mechanism and a case study via thermoacoustic theory. Energy,2023,273: 127282.

[19] Mazzetti, Alessandro, Gianotti Pret, et al. Heat to electricity conversion systems for moon exploration scenarios: A review of space and ground technologies. Acta Astronautica,2019, 156: 162-186.

[20] National Aeronautics and Space Administration, Nuclear Systems Kilopower Overview. https://ntrs.nasa.gov/citations/20160012354 (2016), Accessed 11 May 2025.

[21] National Aeronautics and Space Administration, NASA Space Technology Roadmaps and Priorities: Restoring NASA's Technological Edge and Paving the Way for a New Era in Space. <https://nap.nationalacademies.org/read/13354/chapter/13> (2015), Accessed 31 Aug 2025.

[22] National Aeronautics and Space Administration, Mars Exploration Radioisotope Power and Heating for Mars Surface Exploration.

<https://mars.nasa.gov/internal_resources/790/>, Accessed 31 Aug 2025.

[23] National Aeronautics and Space Administration, A Historical Review of Brayton and Stirling Power Conversion Technologies for Space Applications. https://ntrs.nasa.gov/citations/20070038168 (2007), Accessed 31 Aug 2025.

[24]

[Sandia National Laboratories, Closed Brayton cycle power conversion systems for nuclear reactors. https://www.sandia.gov/research/publications/details/closed-brayton-cycle-power-conversion-systems-for-nuclear-reactors-2006-04-01/ (2006), Acessed 31 Aug 2025.](Sandia National Laboratories, Closed Brayton cycle power conversion systems for nuclear reactors. https://www.sandia.gov/research/publications/details/closed-brayton-cycle-power-conversion-systems-for-nuclear-reactors-2006-04-01/ (2006), Acessed 31 Aug 2025. [25] National Aeronautics and Space Adminstration, A Comparison of Fission Power System Options for Lunar and Mars Surface Applications.)

[[25] National Aeronautics and Space Adminstration, A Comparison of Fission Power System Options for Lunar and Mars Surface Applications.](Sandia National Laboratories, Closed Brayton cycle power conversion systems for nuclear reactors. https://www.sandia.gov/research/publications/details/closed-brayton-cycle-power-conversion-systems-for-nuclear-reactors-2006-04-01/ (2006), Acessed 31 Aug 2025. [25] National Aeronautics and Space Adminstration, A Comparison of Fission Power System Options for Lunar and Mars Surface Applications.)

<https://ntrs.nasa.gov/citations/20060011236> (2006), Accessed 31 Aug 2025.

[26] Mars: Prospective Energy and Material Resources,

<https://link.springer.com/book/10.1007/978-3-642-03629-3> (2009), Accessed 31 Aug 2025.

[27] National Aeronautics and Space Adminstration, Closed Brayton Cycle Power Conversion Unit for Fission Surface Power Phase I Final Report.

https://ntrs.nasa.gov/citations/20100026654 (2010), Accessed 31 Aug 2025.

[28] Weixiong Chen, Meihui Song, Yiran Qian, et al Research and development of helium-xenon Brayton cycle technology: A review. National Science Open, 2024, 3: 191-221.

[29] Xu Chi, Kong Fanli, Yu Dali, et al. Influence of non-ideal gas characteristics on working fluid properties and thermal cycle of space nuclear power generation system. Energy, 2021, 222: 119881.

[30] Baihui Jiang, Yu Ji, Jun Sun, et al. Efficiency and mass optimization for space nuclear power systems with closed Brayton cycle loops. Applied Thermal Engineering, 2024, 253: 123786.

[31] Wenkui Ma, Ping Ye, Yue Gao, et al. Optimization of thermodynamic performance and mass evaluation for MW-class space nuclear reactor coupled with noble gas binary mixtures Brayton cycle. Energy, 2024, 293: 130498.

[32] Wenkui Ma, Ping Ye, Yue Gao, et al. Study on inherent mechanism between thermodynamic performance and mass evaluation of MW-class nuclear powered spacecraft. Thermal Science and Engineering Progress, 2024, 50: 102568.

[33] Zekuan Liu, Lili Wen, Pengyue Liu, et al. Evaluation of the energy system variable operating conditions under the lunar environment. Applied Thermal Engineering, 2025, 269: 126095.

[34] Zijian Sun, Haochun Zhang, QiQi Sun, et al. The influence of lunar environment exposureon characteristics of nuclear energy He–Xe closed Brayton cycle. Acta Astronautica,2024,223: 481-494.

[35] Zijian Sun, Haochun Zhang, QiQi Sun, et al. Evaluation of the high-robustness nuclear power cycle against lunar surface environmental threat. Applied Thermal Engineering, 2024, 253: 123776.

[36] Li Z, Yang XY, Wang J, et al. Off-design performance and control characteristics of space reactor closed Brayton cycle system. Annals of Nuclear Energy, 2019, 128: 318-329.

[37] Yangmao Wu, Simiao Tang, Longxiang Zhu, et al. Transient analysis of megawatt-level space gas-cooled reactor coupled with He-Xe Brayton cycle system. Applied Thermal Engineering, 2025, 260: 124962.

[38] Ming Liu, Pan Wu, Ke Huang, et al. Development and application of safety analysis code for He-Xe cooled space reactor. Nuclear Engineering and Design, 2025, 434: 113914.

[39] Haoyang Liao, Xianbo Wang, Lin Xie, et al. Thermal-hydraulic characteristics analysis of unprotected accident and protection control strategy for helium-xenon cooled reactor system. Energy, 2024, 302: 131763.

[40] Xiangrong Tang, Zongxin Yang, ChuangKan Liu, et al. Design of closed Brayton cycle power generation system for megawatt-scale space nuclear reactor. Energy, 2025, 335: 137978.

[41] Yinke Qi, Xiaofeng Ma, Peixue Jiang, et al. Optimal design and aerodynamic performance analysis of a radial inflow turbine in the helium‒xenon mixture Brayton cycle. Aerospace Science and Technology, 2025, 167: 110699.

[42] Hao Qin, Chenglong Wang, Wenxi Tian, et al. Experimental investigation on flow and heat transfer characteristics of He-Xe gas mixture. International Journal of Heat and Mass Transfer,2022, 192: 122942.

[43] Tianyang Qin, Xinping Yan, Chengqing Yuan, et al. Review of system design and operation control technology of supercritical CO_2_ power cycle. Energy Conversion and Management,2025, 326: 119462.

[44] Zhi Ling, Xuan Wang, Hua Tian, et al. Performance analysis of a underwater power transcritical CO_2_ cycle system prototype. Applied Energy, 2025, 391: 125786.

[45] He, Yaling, Li, Mengjie, Guo, Jiaqi, et al. Advances and development trend of carbon dioxide thermodynamic cycles applied in novel energy systems. Chinese Science Bulletin, 2025, 70(7): 872-887.

[46] Yangfan Ma, Dechao Liu, Jinghan Wang, et al. Thermal-hydraulic performance and optimization of printed circuit heat exchangers for supercritical fluids: A review. Renewable and Sustainable Energy Reviews, 2025, 208: 115051.

[47] Lei Chai, Savvas A. Tassou. A review of printed circuit heat exchangers for helium and supercritical CO_2_ Brayton cycles. Thermal Science and Engineering Progress,2020, 18: 100543.

[48] Kang Yang, Naxin Zhang, Laijie Chen, et al. Performance analysis of a 100 kW S-CO_2_ centrifugal compressor: Design and experimental study. Energy, 2025, 322: 135696.

[49] Huang, Guangdai,Shu,et al. Experiments on a small-scale axial turbine expander used in CO_2_ transcritical power cycle. Applied Energy, 2019, 255: 113853.

[50] Ligeng Li, Hua Tian, Xin Lin, et al. Demonstration of a small-scale power generator using supercritical CO_2_. Carbon Energy, 2024, 6: 273-294.

[51] National Aeronautics and Space Administration, Nuclear Power Concepts and Development Strategies for High-Power Electric Propulsion Missions to Mars,

https://ntrs.nasa.gov/citations/20210016968 (2022), Accessed 31 Aug 2025.

[52] The 8th International Supercritical CO_2_ Power Cycles Symposium, Development of a Dry Gas Seal for high-temperature supercritical carbon dioxide (sCO_2_) turbines,

<https://sco2symposium.com/proceedings2024/33-paper.pdf> (2024), Accessed 31 Aug 2025.

[53] Rautela Mahindra, Mirfarah Motahareh, Silva Christian E, et al. Real-time rapid

leakage estimation for deep space habitats using exponentially-weighted adaptively-refined search. Acta Astronautica, 2023, 203: 385-391.

[54] Yayu M Hew, Kevin J Schillo, Akansha Kumar, et al. Power Management and Distribution System for a Mars Surface Fission Power Reactor. Journal of Nuclear

Engineering and Radiation Science, 2018, 4: 041019.

[55] Qi Xue, Pan Wu, Ming Liu, et al. Thermal hydraulic design and mass optimization of a 100-kWe S-CO_2_ cooled Mars-surface fission reactor system. Annals of Nuclear Energy, 2023, 190: 109893.

[56] Fei-xiang Yuan, Sheng-hui Liu, Min-yun Liu, et al. Design and optimization of space nuclear power system with sCO_2_ Brayton cycle on Mars. Progress in Nuclear Energy, 2024, 176: 105375.

[57] Wu W, Shen J, Kong H, et al. Energy system and resource utilization in space: A state-of-the-art review. The Innovation Energy, 2024, 1(2): 100029.

[58] Becky Sondelski, Greg Nellis. Mass optimization of a supercritical CO_2_ Brayton cycle with a direct cooled nuclear reactor for space surface power. Applied Thermal Engineering,2019, 163: 114299.

[59] Yuzhuo Yang, Lingfeng Shi, Yu Yao, et al. Supercritical CO_2_ Brayton cycle for space exploration: New perspectives base on power density analysis. Energy, 2024, 313: 133772.

[60] Yuzhuo Yang, Yonghao Zhang, Yu Yao, et al. In-situ atmospheric thermoelectric conversion on Mars. Science Bulletin, 2025, 70(13): 2051-2055.

[61] Yansong Han, Shenghui Liu, Yanping Huang, et al. Optimization design of supercritical Brayton cycle with self-supply working fluid based on Martian air for MW-class space surface power. Applied Thermal Engineering, 2025, 269: 126017.

[62] Fangyan Jiang, Yao Fu, Kunlin Cheng, et al. Nuclear power system based on open Brayton cycle for Mars base: configuration comparison and performance assessment. Applied Thermal Engineering, 2025, 279: 127810.

[63] Marcel Veismann, Luis Phillipe Tosi. Evaluation of low-pressure mechanical compression for Martian atmospheric CO_2_ capture: Implications for in-situ resource utilization. Acta Astronaut, 2025, 228: 769-791.

[64] National Aeronautics and Space Administration, Atmospheric Capture on Mars (and Processing), <https://ntrs.nasa.gov/citations/20170001808> (2017), Accessed 31 Aug 2025.

[65] Rapp Donald, [Inglezakis, Vassilis J](https://www.zhizhen.com/s?sw=author%28Inglezakis%2C+Vassilis+J.%3Csup%3E2%3C%2Fsup%3E%29). Mars In Situ Resource Utilization (ISRU) with Focus on Atmospheric Processing for Near-Term Application-A Historical Review and Appraisal. Applied Sciences-Basel, 2024, 14(2): 653.

[66] Donald Rapp. Near-Term NASA Mars and Lunar In Situ Propellant Production: Complexity versus Simplicity. Space Sci. Technol, 2024, 4: 0188.

[67] National Aeronautics and Space Administration, Human Exploration of Mars Design Reference Architecture 5.0, <https://ntrs.nasa.gov/citations/20090040343> (2010), Accessed 31 Aug 2025.

[68] National Aeronautics and Space Administration, Testing and Modeling of the Mars Atmospheric Processing Module, <https://ntrs.nasa.gov/citations/20170009588> (2017), Accessed 31 Aug 2025.

[69] National Aeronautics and Space Administration, Demonstration of Critical Systems for Propellant Production on Mars for Science and Exploration Missions, <https://ntrs.nasa.gov/citations/20130010980> (2013), Accessed 31 Aug 2025.

[70] National Aeronautics and Space Administration, Experimental Design and Preliminary Analysis of a Mars CO_2_ Rapid Cycle Adsorption Pump, <https://ntrs.nasa.gov/citations/20190029042> (2019), Accessed 31 Aug 2025.

[71] Marcel Veismann, Luis Phillipe Tosi. Evaluation of low-pressure mechanical compression for Martian atmospheric CO_2_ capture: Implications for in-situ resource utilization. Acta Astronautica,2025, 228: 769-791.

[72] Forrest E Meyen, Michael H Hecht, Jeffrey A Hoffman, et al. Thermodynamic model of Mars Oxygen ISRU Experiment (MOXIE). Acta Astronautica, 2016, 129: 82-87.

[73] Hecht M, Hoffman J, [Rapp D,](https://www.zhizhen.com/s?sw=author%28Rapp%2C+D.%29) et al. Mars Oxygen ISRU Experiment (MOXIE)Version History. Space Science Reviews, 2021, 217: 9.

[74] [Hinterman Eric](https://www.zhizhen.com/s?sw=author%28Hinterman%2C+Eric%3Csup%3E1%3C%2Fsup%3E%29), [Hoffman Jeffrey A](https://www.zhizhen.com/s?sw=author%28Hoffman%2C+Jeffrey+A%3Csup%3E2%3C%2Fsup%3E%29). Simulating oxygen production on Mars for the Mars Oxygen In-Situ Resource Utilization Experiment. Acta Astronautica, 2020, 170: 678-685.

[75] John B McClean, Jeffrey A Hoffman, Michael H. Hecht, et al. Pre-landing plans for Mars Oxygen In-Situ Resource Utilization Experiment (MOXIE) science operations. Acta Astronautica, 2022, 192: 301-313.

[76] Jeffrey A Hoffman, Michael H Hecht, Donald Rapp, et al. Mars Oxygen ISRU Experiment (MOXIE)-Preparing for human Mars exploration. Science advances, 2022, 8(35): eabp8636.

[77] Plachta D, Stephens J, Johnson W, et al. NASA cryocooler technology developments and goals to achieve zero boil-off and to liquefy cryogenic propellants for space exploration. Cryogenics, 2018, 94: 95-102.

[78] [WL Johnson](https://www.sciencedirect.com/author/55809329400/wesley-l-johnson), DM Hauser, DW Plachta, et al. Comparison of oxygen liquefaction methods for use on the Martian surface.[J]. Cryogenics, 2018, 90: 60-69.

[79] National Aeronautics and Space Administration, Liquefaction and Storage of In-Situ Oxygen on the Surface of Mars, https://ntrs.nasa.gov/citations/20160004210 (2016), Accessed 31 Aug 2025.

[80] Rapp Donald, Hinterman Eric. Adapting a Mars ISRU System to the Changing Mars Environment. Space Sci. Technol, 2023, 3:41.

[81] Jeffrey A Hoffman, Eric R Hinterman, Michael H Hecht, et al. 18 Months of MOXIE (Mars oxygen ISRU experiment) operations on the surface of Mars - Preparing for human Mars exploration. Acta Astronautica,2023, 210: 547-553.

[82] National Aeronautics and Space Administration, Human Exploration of Mars: The Reference Mission of the NASA Mars Exploration Study Team,

<https://ntrs.nasa.gov/citations/19980037039> (1997), Accessed 31 Aug 2025.

[83] Shah Saud Alam, Christopher Depcik, Sindhu Preetham Burugupally, et al. Thermodynamic modeling of in-situ rocket propellant fabrication on Mars. iScience, 2022, 25: 104323.

[84] Chen Hao, Sarton du Jonchay, et al. Integrated in-situ resource utilization system design and logistics for Mars exploration. Acta Astronautica,2020, 170: 80-92.

[85] Daniel Vazquez Pombo. A Hybrid Power System for a Permanent Colony on Mars. Space Sci. Technol, 2021, 2021: 9820546.

[86] Andrew Abercromby, David Baumann, Debbie Berdich, et al. NASA's top human system research and technology needs for Mars. Acta Astronautica,2025, 228: 931-939.

[87] National Aeronautics and Space Administration, NASA’s Moon to Mars (M2M) Transit Habitat Refinement Point of Departure Design,

<https://ntrs.nasa.gov/citations/20220014975> (2023), Accessed 31 Aug 2025.

[88] Liuqing Yang, Ce Zhang, Xiwen Yu, et al. Extraterrestrial artificial photosynthetic materials for in-situ resource utilization. National Science Review,2021,8: 108-133.

***Thermodynamic analysis for proposed design framework***

***(i) Combined power and heating production***

The sustainable and long-term thermal energy supplement is necessary for Mars bases. The internal temperature of the nuclear reactor is excessively high (~1000 K), and directly utilizing for heating would result in a significant thermal energy waste. The COP of electric heaters is less than 1, and energy will flow from high-quality (electricity) to low-quality (thermal). The abundant low-temperature waste heat resources from the Martian air heat-to-electricity conversion system are used for thermal energy supply, which enhances the primary energy efficiency. The internal energy flowing of the integrated system is shown in **Fig. S1 (a)**.

The thermodynamic T-S diagram of integrated system is shown in **Fig. S1 (b).** The efficiency of the heat-to-electricity module, which was independent before integration, can be expressed as equation (S1).

$\eta_{0}=\frac{P_{HTEC}-P_{medium}}{Q_{reactor}}$ (S1)

Where, *P_HTEC_* is the net output power of heat-to-electricity module. *P_medium_* is the power consumption of Martian atmosphere capture. *Q_reactor_* is the thermal energy input by nuclear reactor.

The heat-to-electricity conversion can cyclically utilize Martian air, as the power consumption caused by leakage and replenishment is much lower than its output power (*P_HTEC_* >> *P_medium_*). The efficiency can be approximately expressed as equation (S2).

$\eta_{0}=\frac{P_{HTEC}}{Q_{reactor}}=\frac{S_{1234}}{S_{b23d}}$ (S2)

Where, the area *1-2-3-4* (S*_1234_*) represents the net output power of heat-to-electricity module. The area *b-2-3-d* (*S_b23d_*) represents the thermal energy input by nuclear reactor.

The overall efficiency of the integrated system can be expressed as equation (S3). Clearly, the utilization of waste heat can improve the overall efficiency.

$\eta=\frac{P_{HTEC}+Q_{heating}}{Q_{reactor}}=\frac{S_{1234}+n\cdot S_{a15c}}{S_{b23d}}$ (S3)

Where, the area *a-1-5-c* (*S_a15c_*) represents the available low-grade waste heat recovery capacity. *n* represents the actual utilization ratio (0~1).

***
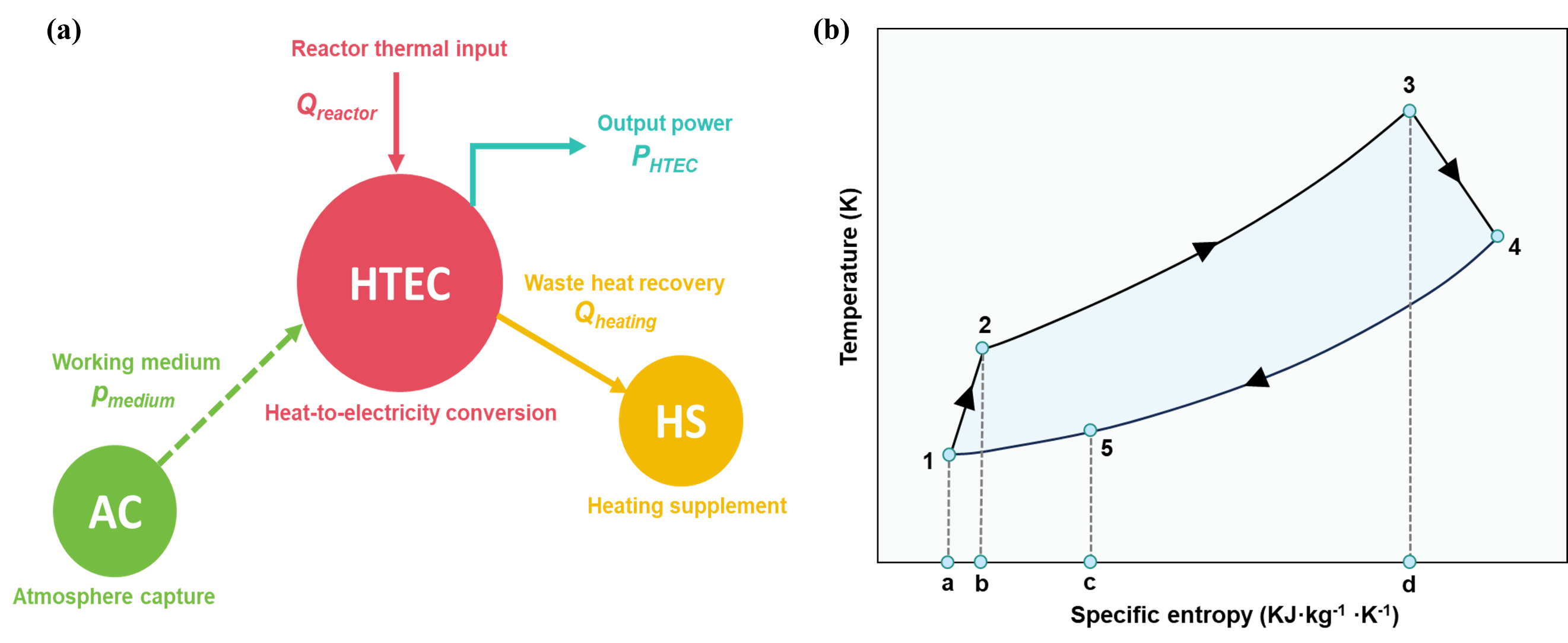
***

**Fig. S1** Schematic of the principle of in-situ combined power and heating production. (a) energy flowing (b) thermodynamic T-S diagram

***(ii) Combined power and SOEC module***

The working medium of the thermoelectric conversion module is the same as the reactants in the SOEC module. The excellent thermodynamic properties and electrochemical characteristics of Martian air lay a foundation for both processes coupling to enhance efficiency and enable in-situ life-support resources transformation. The suitable temperature and pressure for the SOEC are close to exhaust gas (after expansion) state of the heat-to-electricity conversion (**Fig. S2 (b)**). The internal energy flowing of the integrated system is shown in **Fig. S2 (a)**. Next, the changes in energy efficiency for the two subsystems will be discussed separately.

It can be seen from **Fig. S2 (b)** that the reactants for the SOEC module are sourced from the working medium in the high-temperature waste heat region of the heat-to-electricity conversion system. The lost working medium (equivalent to the reactants molar flow rate in SOEC) is replenished by the atmospheric capture system, and the efficiency of heat-to-electricity conversion module within integrated system can be expressed as equation (S4). In other words, it can be said that the thermoelectric conversion system only loses a portion of the high-temperature waste heat, with no impact on the power generation efficiency. Because the increase in atmospheric capture power consumption is caused by the need for reactant supply to the SOEC module, it is not accounted for within the heat-to-electricity conversion module.

$\eta=\frac{P_{HTEC}}{Q_{reactor}}=\frac{S_{1234}}{S_{b23d}}$ (S4)

Where, the area *1-2-3-4* (S*_1234_*) represents the net output power of heat-to-electricity module. The area *b-2-3-d* (*S_b23d_*) represents the thermal energy input by nuclear reactor.

The thermodynamic T-S diagram of Martian air SOEC module within integrated system is shown in **Fig. S2 (c)**. Although the input electricity in the SOEC cannot be directly represented in the T-S indicator diagram, it can be indirectly expressed by the area difference corresponding to the two thermodynamic processes. After the Martian air (CO_2_, N_2_, Ar) enter the cathode, they are electrolyzed to generate products (CO, O_2_, N_2_, Ar), with the electrolysis being an endothermic process where the temperature decreases from T_1_' to T_2_'.

Assuming that the products (CO, O_2_, N_2_, Ar) are completely combusted in the burner, with all the chemical energy being released as thermal energy, the temperature of the exhaust gas will reach T_3_'. The area *a-c-f-h* (*S_acfh_*) represents the chemical energy released form the products. Base on first law of thermodynamics, the area a-b-g-h (*S_abgh_*) represents the electrical energy consumed in the electrolysis process. The area b-d-e-g (*S_bdeg_*) represents the heat absorption capacity by reactants form initial temperature (T_0_) to reaction temperature (T_1_'). the efficiency of independent SOEC module can be expressed as equation (S5). The power consumption for capturing Martian atmosphere is not calculated in the efficiency equation, and this value is generally fixed.

$\eta_{0}=\frac{E_{products}}{P_{electrolysis}+Q_{heating}}=\frac{S_{acfh}}{S_{abgh}+S_{b\deg}}$ (S5)

Where, *E_products_* represents the chemical energy of products. *P_electrolysis_* represents the electrical energy consumed in the electrolysis process. *Q_heating_* is the heat absorption capacity by reactants form initial temperature to reaction temperature.

The high-temperature exhaust Martian air from heat-to-electricity conversion module is directly used as the SOEC reactant, which avoids the additional energy consumption in the heating process. The efficiency of SOEC module within integrated system can be expressed as equation (S6). As the scale of SOEC increases, the energy-saving effect becomes more significant, especially when producing fuel for the rocket return. Moreover, the footprint and mass of the SOEC module can be further reduced, eliminating the auxiliary components (recuperator, heater and pre-compressor), as shown in **Fig. S2 (d)**.

$\eta==\frac{S_{acfh}}{S_{abgh}}$ (S6)


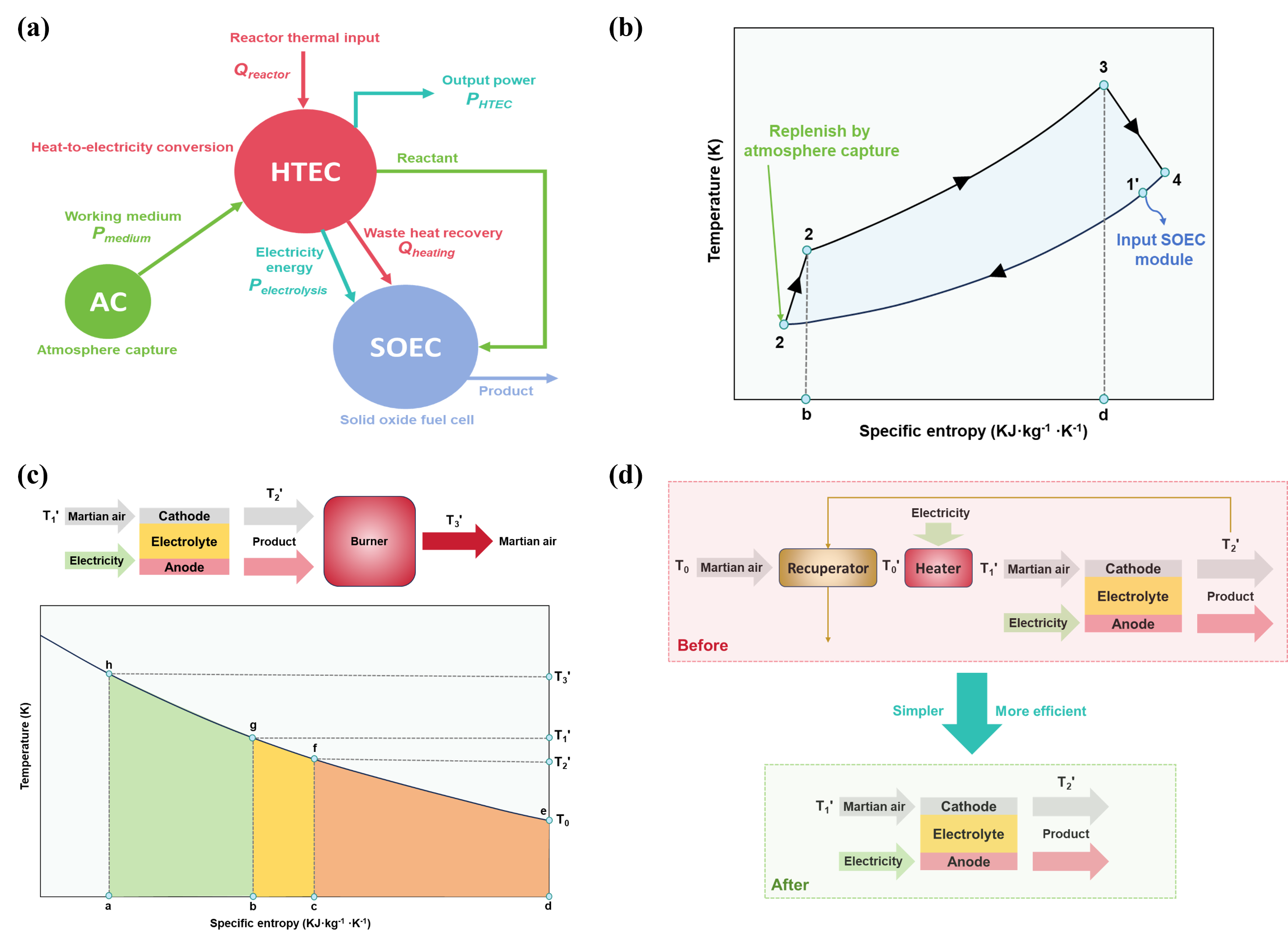


**Fig. S2** Schematic of the principle of in-situ combined power and SOEC module. (a) energy flowing (b) thermodynamic T-S diagram of heat-to-electricity conversion (c) thermodynamic T-S diagram of SOEC module (d) the structure of the SOEC module before and after integration.

***Potential assessment for proposed design framework***

1. ***Energy requirements for early manned Mars missions***

**Table S1.** Detailed propellant and survival requirements for early manned Mars mission.

| Type | Value |
| --- | --- |
| Liquid oxygen (MAV propellant) | 30804 kg (Single mission) |
| Methane (MAV propellant) | 9408 kg (Single mission) |
| ISRU allowed time | 10080 h |
| In-situ oxygen production rate | ≥3.06 kg/h |
| Average power levels for Mars habitat | 20000 W |
| Heating requirements for Mars habitat | 3300 W |

* The propellant consumption of the Mars ascent vehicle (MAV) and energy data for the habitat are sourced from NASA's technical reports.

MAV date: <https://ntrs.nasa.gov/api/citations/20160006401/downloads/20160006401.pdf>

Habitat date: https://[ntrs.nasa.gov/api/citations/20170002219/downloads/20170002219.pdf](https://ntrs.nasa.gov/api/citations/20170002219/downloads/20170002219.pdf)

* To increase from a crew of four to a crew size of six, the requirement is multiplied by a factor of 1.356, derived from the ratio of propellant required between MAV designs for four-person and six-person crews in Mars Design Reference Architecture 5.0.

Mars Design Reference Architecture 5.0: <https://ntrs.nasa.gov/citations/20090040343>

* This study uses the requirements of six-persons as an example. Based on Mars Design Reference Architecture 5.0, early crewed Mars missions focus on atmospheric resource utilization, so the case design framework results presented later do not involve the more complex Sabatier reaction.

1. ***In-situ liquid-oxygen production***





**Fig. S3**. Internal energy and material flow for proposed in-situ oxygen production plant. Martian atmosphere as a working medium for and thermodynamic processes and chemical transformation. The above design framework only considers in-situ oxygen production, and the system can prepare the required oxygen (30804 kg, operating 10080h) before humans arrive on Mars.

*In the case study, the capture of the Martian atmosphere uses a cryogenic trapping scheme.





**Fig. S4**. Internal energy and material flow for in-situ oxygen production plant powered by solar arrays or NASA Kilopower (Nuclear Stirling Engine: A single unit has power options of 1, 3, 5, 7, and 10 kW). Both solar arrays and Kilopower are potential technologies developed by NASA for large-capacity power generation on Mars, making them ideal candidates for powering Martian missions.

Solar arrays data: <https://ntrs.nasa.gov/citations/20170006908>

Kilopower data: <https://ntrs.nasa.gov/citations/20160012354>

*In the case study, the capture of the Martian atmosphere uses a cryogenic trapping scheme.

**
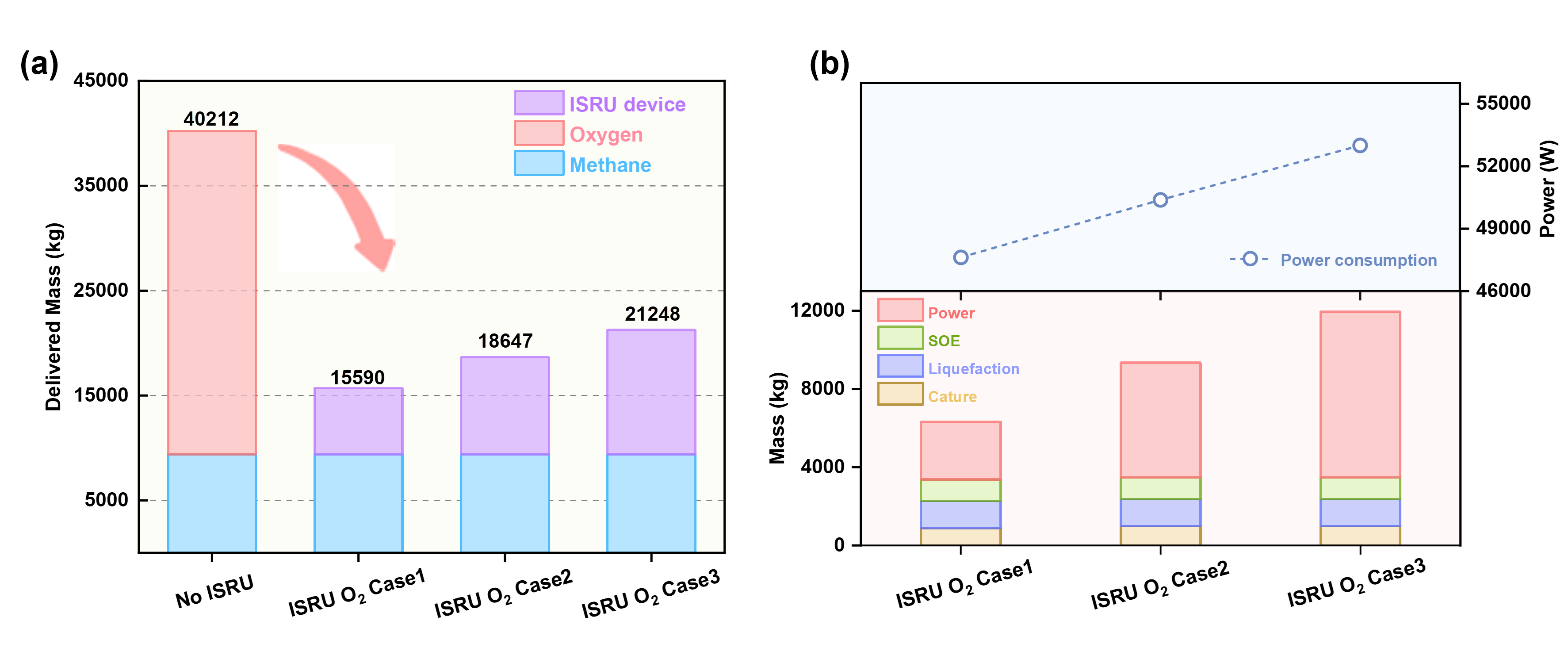
**

**Fig. S5**. Technological competitiveness of the in-situ oxygen production plant for manned Mars missions. (a) Delivered propellant mass from Earth to Mars for MAV. (b) Mass distribution and overall power consumption. Case 1: Proposed design framework in this study. Case 2: Comparison system powered by solar arrays. Case 3: Comparison system powered by Kilopower.

**Table S2.** Power and mass of key components in the above in-situ oxygen production plant.

| **Components** | | | | **Case 1** | | **Case 2** | | | | **Case 3** | | |
| --- | --- | --- | --- | --- | --- | --- | --- | --- | --- | --- | --- | --- |
|  |  |  |  | **In-situ power generation** | | **Solar array** | | | | **Stirling engine (Kilopower)** | | |
|  |  |  |  | Power (W) | Mass (kg) | Power | | | Mass | Power | | Mass |
| **Capture system** | | | | 18588 | 873.9 | 22062 | | | 873.9 | 22062 | | 873.9 |
| 1 | Blower | | | 120.6 | 6.2 | 120.6 | | | 6.2 | 120.6 | | 6.2 |
| 2 | Valve | | | 9 | 12.6 | 9 | | | 12.6 | 9 | | 12.6 |
| 3 | Cryochamber | | | 2449.8* | 288.9 | 2449.8* | | | 288.9 | 2449.8* | | 288.9 |
| 4 | Cryocooler | | | 15605 | 418.6 | 15605 | | | 418.6 | 15605 | | 418.6 |
| 5 | Heater (In chamber) | | | 2853.5 | -- | 6327.1 | | | -- | 6327.1 | | -- |
| 6 | Radiator | | | 17275* | 147.6 | 17275* | | | 147.6 | 17275* | | 147.6 |
| **Power system** | | | | 47624(Output) | 2943.9 | 50385(Output) | | | 5869.7 | 53000(Output) | | 8471 |
| 1 | Rotating unit | | | 47624 | 239.7 | -- | | | | -- | | |
| 2 | Space reactor | | | 246150* | 574.2 | -- | | | | -- | | |
| 3 | Nuclear shielding | | | -- | 462.0 | -- | | | | -- | | |
| 4 | Recuperator | | | 510920* | 352.9 | -- | | | | -- | | |
|  | 4.1 | | Core | 510929* | 244.0 | -- | | | | -- | | |
|  | 4.2 | | Shell | -- | 76.0 | -- | | | | -- | | |
|  | 4.3 | | Insulating material | -- | 32.9 | -- | | | | -- | | |
| 5 | Cooler | | | 196020* | 301.3 | -- | | | | -- | | |
|  | 5.1 | | Core | 196020* | 207.8 | -- | | | | -- | | |
|  | 5.2 | | Shell | -- | 65.3 | -- | | | | -- | | |
|  | 5.3 | | Insulating material | -- | 28.2 | -- | | | | -- | | |
| 6 | Radiator | | | 196020* | 711.7 | -- | | | | -- | | |
| 7 | Connecting pipe | | | -- | 302.0 | -- | | | | -- | | |
|  | 7.1 | | Material | -- | 164.5 | -- | | | | -- | | |
|  | 7.2 | | Valve | -- | 14 | -- | | | | -- | | |
|  | 7.3 | | Insulating material | -- | 123.5 | -- | | | | -- | | |
| 8 | Solar arrays | | | -- | | 50385 | 3930.3 | | | -- | | |
| 9 | Lithium-ion battery | | | -- | | (688kWh) | 1939.4 | | | -- | | |
| 10 | Kilopower | | | -- | | 47000 | 7422 | | | 53000 | 8471 | |
|  | 10.1 | | 10kW-unit | -- | | -- | | | | 5×10000 | 5×1544 | |
|  | 10.2 | | 7kW-unit | -- | | -- | | | | -- | 1246 | |
|  | 10.3 | | 5kW-unit | -- | | -- | | | | -- | 1011 | |
|  | 10.4 | | 3kW-unit | -- | | -- | | | | 1×3000 | 1×751 | |
| **Solid oxide electrolysis** | | | | 16441 | 1103.8 | 16441 | | 1103.8 | | 16441 | | 1103.8 |
| 1 | Cell stacks | | | 16441 | 281.8 | 16441 | | 281.8 | | 16441 | | 281.8 |
|  | 1.1 | | Electrolysis | 14972 | -- | 14972 | | -- | | 14972 | | -- |
|  | 1.2 | | Heating preservation | 1469 | -- | 1469 | | -- | | 1469 | | -- |
| 2 | Inner box | | | -- | 218.9 | -- | | 218.9 | | -- | | 218.9 |
| 3 | Outer box | | | -- | 603.1 | -- | | 603.1 | | -- | | 603.1 |
| **Oxygen liquefaction** | | | | 6843.5 | 1391.6 | 6843.5 | | 1391.6 | | 6843.5 | | 1391.6 |
| 1 | Storage system | | | -- | 1149.1 | -- | | 1149.1 | | -- | | 1149.1 |
|  | 1.1 | Storage tank | | -- | 602.7 | -- | | 602.7 | | -- | | 602.7 |
|  | 1.2 | Insulating material | | -- | 25.4 | -- | | 25.4 | | -- | | 25.4 |
|  | 1.3 | Vacuum jacket | | -- | 154.0 | -- | | 154.0 | | -- | | 154.0 |
|  | 1.4 | Supporting structure | | -- | 301.3 | -- | | 301.3 | | -- | | 301.3 |
|  | 1.5 | Pipe system | | -- | 65.7 | -- | | 65.7 | | -- | | 65.7 |
| 2 | Cryocooler | | | 6843.5 | 159.9 | 6843.5 | | 159.9 | | 6843.5 | | 159.9 |
| 3 | Radiator | | | 6989.0* | 81.4 | 6989.0* | | 81.4 | | 6989.0* | | 81.4 |
| 4 | Radiator for precooler | | | 715.8* | 1.1 | 715.8* | | 1.1 | | 715.8* | | 1.1 |
| **Gas regulator** | | | | 988.2 | -- | -- | | | | -- | | |
| **Total** | | | | 42861 | 6313.2 | 45347 | | 9239.0 | | 41054 | | 11840.0 |

The symbol (*) above indicates that this value represents thermal energy.

The rated power generation capacity of the power system considers a 10% redundancy.

1. ***In-situ energy station***





**Fig. S6**. Internal energy and material flow for proposed in-situ energy station. The above design framework both considers in-situ oxygen production (30804kg, 10080h) and habitat requirement.

*In the case study, the capture of the Martian atmosphere uses a cryogenic trapping scheme.

**Table S3**. Power and mass of key components in the proposed in-situ energy station.

| Components | | | | In-situ energy station. | |
| --- | --- | --- | --- | --- | --- |
|  |  |  |  | Power (W) | Mass (kg) |
| **Capture system** | | | | 18588 | 873.9 |
| 1 | Blower | | | 120.6 | 6.2 |
| 2 | Valve | | | 9 | 12.6 |
| 3 | Cryochamber | | | 2449.8* | 288.9 |
| 4 | Cryocooler | | | 15605 | 418.6 |
| 5 | Heater (In chamber) | | | 2853.5 | -- |
| 6 | Radiator | | | 17275* | 147.6 |
| **Power system** | | | | 69846(Output) | 3748.5 |
| 1 | Rotating unit | | | 69846 | 313.4 |
| 2 | Space reactor | | | 361000* | 598.3 |
| 3 | Nuclear shielding | | | -- | 508.1 |
| 4 | Recuperator | | | 749320* | 499.4 |
|  | 4.1 | | Core | 749320* | 362.2 |
|  | 4.2 | | Shell | -- | 95.8 |
|  | 4.3 | | Insulating material | -- | 41.4 |
| 5 | Cooler | | | 284180* | 422.1 |
|  | 5.1 | | Core | 284180* | 304.2 |
|  | 5.2 | | Shell | -- | 82.3 |
|  | 5.3 | | Insulating material | -- | 35.6 |
| 6 | Radiator | | | 212640* | 1077 |
| 7 | Connecting pipe | | | -- | 371.2 |
|  | 7.1 | | Material | -- | 223.9 |
|  | 7.2 | | Valve | -- | 14 |
|  | 7.3 | | Insulating material | -- | 133.3 |
| 8 | Heater | | | 2853.5* | -- |
| **Solid oxide electrolysis** | | | | 16441 | 1103.8 |
| 1 | Cell stacks | | | 16441 | 281.8 |
|  | 1.1 | Electrolysis | | 14972 | -- |
|  | 1.2 | Heating preservation | | 1469 | -- |
| 2 | Inner box | | | -- | 218.9 |
| 3 | Outer box | | | -- | 603.1 |
| **Oxygen liquefaction** | | | | 6843.5 | 1391.6 |
| 1 | Storage system | | | -- | 1149.1 |
|  | 1.1 | Storage tank | | -- | 602.7 |
|  | 1.2 | Insulating material | | -- | 25.4 |
|  | 1.3 | Vacuum jacket | | -- | 154.0 |
|  | 1.4 | Supporting structure | | -- | 301.3 |
|  | 1.5 | Pipe system | | -- | 65.7 |
| 2 | Cryocooler | | | 6843.5 | 159.9 |
| 3 | Radiator | | | 6989.0* | 81.4 |
| 4 | Radiator for precooler | | | 756.9* | 1.1 |
| **Gas regulator** | | | | 988.2 | -- |
| **Mars Habitat** | | | | 3300* and 20000 | 43000 |
| **Methane (from Earth)** | | | | -- | 9408 (Single mission) |
| **Total** **launch mass** | | | | -- | 59525.8 |

The rated power generation capacity of the power system considers a 10% redundancy.

The symbol (*) above indicates that this value represents thermal energy.

The Sabatier reaction was not taken into consideration for the initial mission.

**Table S4.** Power and mass of the Martian energy station without ISRU.

| Components | Power (W) | Mass (kg) |
| --- | --- | --- |
| Mars Habitat | 3300* and 20000 | 43000 |
| Power system (Kilopower) | 25000 | 4099 |
| Oxygen | -- | 30804 (Single mission) |
| Methane | -- | 9408 (Single mission) |
| **Total launch mass** | -- | 87311 |

***Modeling method and MARLAB code***

The calculation method for the Mars atmosphere capture system is based on the literature ^[A1, A2, A3]^. The calculated energy consumption for collecting Martian atmosphere per unit (6385.4 J/g) is close to the theoretical result ^[A4]^. The reverse Brayton refrigeration cycle is selected for low-temperature cooling in space (cooling capacity>500W), with system design and working fluid choices based on the literature ^[A2, A5, A6, A7]^. The heat-to-electricity conversion model, including the heat exchanger, rotating machinery, connecting pipes, and radiators, was thoroughly introduced and validated in our previous research ^[A8]^. The weight model of the space nuclear reactor is sourced from Sandia Laboratories (RSMASS-D-Models) ^[A9]^. The space radiator is estimated under extreme conditions (sky temperature~260K), with thermal emissivity based on NASA's experimental data ^[A10]^.

The efficiency of the rotating machinery is crucial for the heat-to-electricity conversion system, with NASA's predictions serving as the benchmark for similar scale ^[A11, A12]^. The parameter design and component weight assessment of the solid oxide electrolysis (SOE) are based on a series of original works by the NASA's MOXIE team ^[A2, A13, A14, A15]^, with the reaction kinetics model referenced from the literature ^[A16]^. **Fig. S7** presents the model validation results for SOE, and the model calculation results are accurate. Large amounts of oxygen need to be stored in liquid form, with the liquefaction model referencing the NASA's research design ^[A17, A18]^, while also considering the heating loss in the pipelines and tanks.

The weight and performance degradation data (loss 0.14% per sol) of the solar arrays on Mars are referenced from NASA's report ^[A19]^, and the performance prediction model for the solar arrays is based on the literature ^[A20]^. On Mars, sky temperature, wind speed, and solar flux influence the temperature of solar cells, which in turn affects their performance ^[A21]^. This study incorporates the effects of Martian climate conditions for solar arrays ^[A22]^. **Fig. S8** presents the model validation results for solar arrays. The performance of the Kilopower unit (Nuclear Stirling engine) is based on the results from NASA's publicly available report ^[A23]^.

***Coding method reference:***

[A1] Stanley O. Starr, et al (2020). https://doi.org/10.1016/j.pss.2019.104824.

[A2] Hinterman E (2022). https://dspace.mit.edu/handle/1721.1/145095.

[A3] [Anne J. Meier](https://arc.aiaa.org/doi/10.2514/6.2018-5172), et al (2018). <https://doi.org/10.2514/6.2018-5172>.

[A4] Zhang et al (2022). 10.3969/j.issn.1000-6516.2022.06.009.

[A5] Weibo Chen, et al (2024). https://doi.org/10.1016/j.cryogenics.2024.103877.

[A6] Aman Kumar Dhillon, et al (2021). https://doi.org/10.1016/j.cryogenics.2021.103262.

[A7] Alessandro Biglia, et al (2017). http://dx.doi.org/10.1016/j.ijrefrig.2017.05.022.

[A8] Yuzhuo Yang, et al (2025). https://doi.org/10.1016/j.scib.2025.04.013.

[A9] Albert C. Marshall (1997). <https://doi.org/10.2172/560867>

[A10] D. Keith Hollingsworth, et al (2006). 10.1016/j.applthermaleng.2006.02.034

[A11] Steven A. Wright, et al (2006). https://www.sandia.gov/research/publications/details/closed-brayton-cycle-power-conversion-systems-for-nuclear-reactors-2006-04-01/ (2006).

[A12] Chen et al (2024). https://doi.org/10.1360/nso/20230089

[A13] Meyen, Forrest Edward (2017). https://dspace.mit.edu/handle/1721.1/112456

[A14] Forrest E. Meyen, et al (2016). http://dx.doi.org/10.1016/j.actaastro.2016.06.005

[A15] Donald Rapp (2023). https://doi.org/10.34133/space.0041

[A16] Zhang et al (2013). http://dx.doi.org/10.1016/j.ijhydene.2013.05.155

[A17] W.L. Johnson, et al (2018). https://doi.org/10.1016/j.cryogenics.2017.12.008

[A18] Daniel M. Hauser, et al. https://ntrs.nasa.gov/citations/20160004210

[A19] FISO Working Group (2017). https://ntrs.nasa.gov/citations/20170006908

[A20] Shi et al (2020). 10.15982/j.issn.2096-9287.2020.20200042

[A21] Alfonso Delgado-Bonal, et al (2015). http://dx.doi.org/10.1016/j.solener.2015.04.035

[A22] G.M. Martínez, et al (2017). 10.1007/s11214-017-0360-x

[A23] Don Palac, et al (2016). https://ntrs.nasa.gov/citations/20160012354


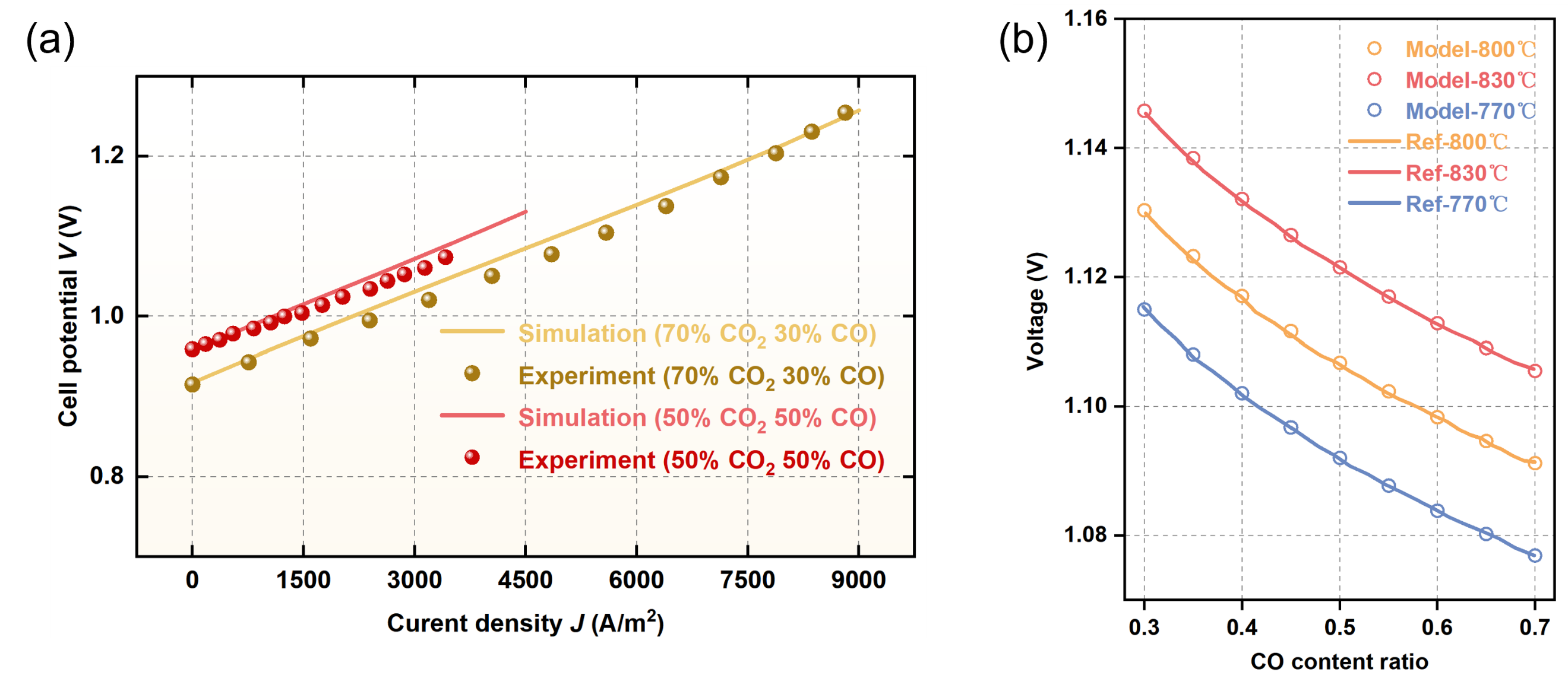


**Fig. S7**. Validation of the solid oxide electrolysis model. (a) Operating voltage at different temperatures and current densities ^[A16]^. (b) Nernst potential for the carbonization reaction (adverse reaction) ^[A13]^.

**
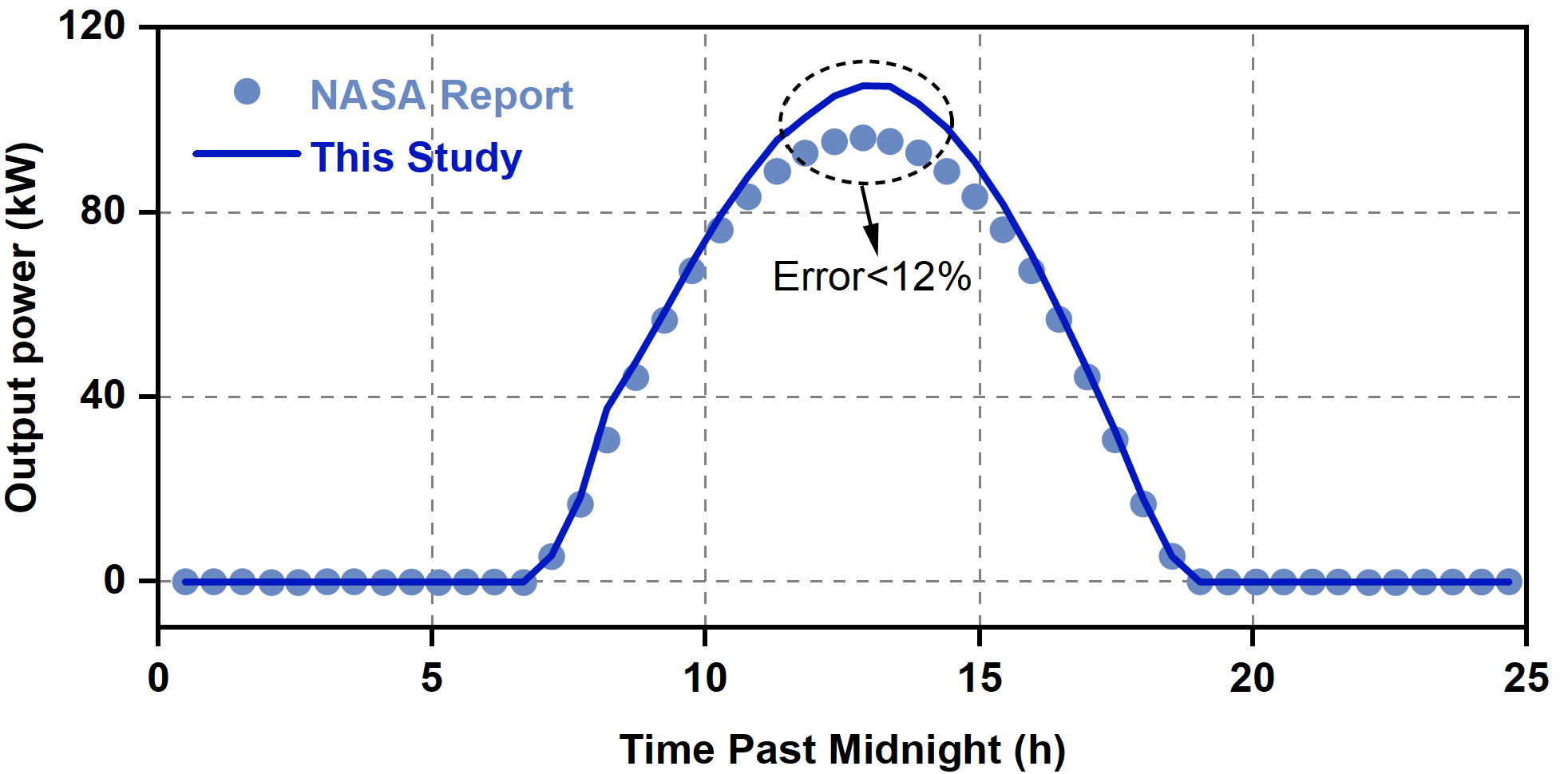
**

**Fig. S8**. Validation of the solar arrays model ^[A19]^.

***MATLAB Code: Proposed in-situ oxygen production plant (Fig S3)***

%% Mission objective

Polsgrove_Crew=30804; % Target oxygen production volume, kg

ISRU_Time_Allowed=14; % ISRU Allowed Time, month

mO2_require=Polsgrove_Crew/(ISRU_Time_Allowed*30*24); % Oxygen production rate, kg/h

U_SOE=0.8; % The gas utilization rate in the SOE system

%%%%%%

%% Martian atmosphere capture system

% Basic parameter input

eff_cryocooler_capture=0.7;% Capturing efficiency，Stanley O. Starr，et al.

fluid1='CO2';

fluid2='NITROGEN';

fluid3='argon';

ratio=[0.968,0.017,0.015]; % Composition of atmosphere, CO2,N2,Ar

P_atmosphere=600*10^(-3); % Atmospheric average pressure, kPa

T_atmosphere=260; % Atmospheric temperature, K (Conservative conditions)

rho_atmosphere=refpropm('D','T',T_atmosphere,'P',P_atmosphere,fluid1,fluid2,fluid3,ratio); % Atmospheric density, kg/m3

atmosphere_speed=3;% Average wind speed, m/s

eff_blower=0.6;% Blower efficiency

eff_ISRU_heater=0.9;% Heater efficiency

P0_blower=16800; % The power consumption of blower, J/kg, Eric Daniel

Hinterman et al.

M0_blower=0.4; % The unit mass of blower, kg/(kg/h), Eric Daniel

Hinterman et al.

heat_loss_cryocooler=0.85; % Heating loss in cryochamber

T_cryo=148; % CO2 deposition temperature, K

M0_valve_single=1.4; % Valve unit mass, kg

P0_valve_single=1; % the power consumption of valve unit, W

% Power consumption calculation during the blowing stage

x_CO2=ratio(1); % The mass fraction of CO2 in the atmosphere

Mm_CO2=0.044; % Molar mass of CO2，kg/mol

Mm_O2=0.032; % Molar mass of O2，kg/mol

m_atmosphere_blower=((((mO2_require/3600)/Mm_O2)*2)/(eff_cryocooler_capture*U_SOE*x_CO2))*Mm_CO2; % Mass flow rate in blower, kg/s. Multiplying by 2 takes into account the stoichiometric ratio of the CO_2_ → CO + 0.5O_2_ reaction

Power_blower=(m_atmosphere_blower*P0_blower)/eff_blower; % Total power consumption in blower, W

M_blower=(m_atmosphere_blower*3600)*M0_blower; % Total mass of blower, kg

Num_Cryochamber=4; % The number of cryogenic chamber M_valve=M0_valve_single*(2*Num_Cryochamber+1); % The number of valve in chamber

Power_valve=P0_valve_single*(2*Num_Cryochamber+1); % The power consumption of valve

% Upstream cooling power calculation

H_deposition_CO2=591; % Latent heat of CO2 deposition, J/g

T_aver_cryo=(T_atmosphere+T_cryo)*0.5; % Mean temperature, K

Cp_atmosphere_aver_cryo=refpropm('C','T',T_aver_cryo,'P',P_atmosphere,fluid1,fluid2,fluid3,ratio); % The average specific heat of the sensible heat cooling process, J/(kg K)

Q_cool_atmosphere=m_atmosphere_blower*Cp_atmosphere_aver_cryo*(T_atmosphere-T_cryo); % Average (sensible heat part) cooling capacity, W

m_ISRU_CO2=(m_atmosphere_blower*x_CO2*eff_cryocooler_capture); % The extraction rate of CO2 from Martian atmosphere, kg/s

Q_deposition_atmosphere=(m_ISRU_CO2*H_deposition_CO2)*1000; % Average (Latent heat part) cooling capacity, W

Q_cryo_atmosphere=(Q_deposition_atmosphere+Q_cool_atmosphere)/heat_loss_cryocooler; % The cooling capacity of the cryocooler, W

% Heating within a fixed volume to cause CO2 to vaporize (boost pressure)

P_ISRU_CO2=600; % Gas supply pressure, kPa

T_ISRU_CO2=323; % Gas supply temperature, K

T_aver_heating=(T_ISRU_CO2+T_cryo)*0.5;

Cp_CO2_heating_aver=refpropm('C','T',T_aver_heating,'P',P_ISRU_CO2,'CO2');

Q_heating_ISRU=m_ISRU_CO2*Cp_CO2_heating_aver*(T_ISRU_CO2-T_cryo); % Average (sensible heat part) heating capacity, W

Q_sublimation_ISRU=m_ISRU_CO2*H_deposition_CO2*1000; % Average (Latent heat part) heating capacity, W

Q_heating_pureCO2=(Q_sublimation_ISRU+Q_heating_ISRU)/heat_loss_cryocooler;% Total required heating capacity for heater, W

Power_heater_ISRU=Q_heating_pureCO2/eff_ISRU_heater; % The power consumption of heater, W

% Cryocooler for capturing CO2

delta_T_reBrayton_CO2cooler_min=5; % Minimum heat transfer temperature difference

eff_reBrayton_CO2cooler_comp=0.7; % compressor isentropic efficiency

eff_reBrayton_CO2cooler_turbine=0.7; % Turbine isentropic efficiency

eff_reBrayton_CO2cooler_R=0.95; % The efficiency of recuperator

delta_P_reBrayton_CO2cooler_C=0.02; % Pressure loss ratio in cooler

delta_P_reBrayton_CO2cooler_R=0.02; % Pressure loss ratio in recuperator

delta_P_reBrayton_CO2cooler_rad=0.02; % Pressure loss ratio in radiator

PR_reBrayton_CO2cooler=2.5; % pressure ratio

% CO2 cold-head side

CO2cooler_gas='NITROGEN'; % Working fluid

P1_reBrayton_CO2cooler=400; %Turbine outlet pressure, kPa

T2_reBrayton_CO2cooler=T_cryo-delta_T_reBrayton_CO2cooler_min; %cold-head outlet temperature, kPa

P2_reBrayton_CO2cooler=P1_reBrayton_CO2cooler*(1-delta_P_reBrayton_CO2cooler_C); %cold-head outlet pressure, kPa

h2_reBrayton_CO2cooler=refpropm('H','T',T2_reBrayton_CO2cooler,'P',P2_reBrayton_CO2cooler,CO2cooler_gas);

% Pressure parameters

P3_reBrayton_CO2cooler=P2_reBrayton_CO2cooler*(1-delta_P_reBrayton_CO2cooler_R); %compressor inlet pressure, kPa

P4_reBrayton_CO2cooler=P3_reBrayton_CO2cooler*PR_reBrayton_CO2cooler; %compressor outlet pressure, kPa

P5_reBrayton_CO2cooler=P4_reBrayton_CO2cooler*(1-delta_P_reBrayton_CO2cooler_rad); %radiator outlet pressure, kPa

P6_reBrayton_CO2cooler=P5_reBrayton_CO2cooler*(1-delta_P_reBrayton_CO2cooler_R); %turbine inlet pressure, kPa

% Calculation of the recuperator

T5_reBrayton_CO2cooler=300; %radiator outlet temperature, K

h5_reBrayton_CO2cooler=refpropm('H','T',T5_reBrayton_CO2cooler,'P',P5_reBrayton_CO2cooler,CO2cooler_gas);

T6_reBrayton_CO2cooler=T5_reBrayton_CO2cooler-(eff_reBrayton_CO2cooler_R*(T5_reBrayton_CO2cooler-T2_reBrayton_CO2cooler)); % turbine inlet temperature

h6_reBrayton_CO2cooler=refpropm('H','T',T6_reBrayton_CO2cooler,'P',P6_reBrayton_CO2cooler,CO2cooler_gas);

h3_reBrayton_CO2cooler=(h5_reBrayton_CO2cooler-h6_reBrayton_CO2cooler)+h2_reBrayton_CO2cooler; % energy balance

T3_reBrayton_CO2cooler=refpropm('T','P',P3_reBrayton_CO2cooler,'H',h3_reBrayton_CO2cooler,CO2cooler_gas); %compressor inlet temperature, K

% compression and expansion process s3_reBrayton_CO2cooler=refpropm('S','P',P3_reBrayton_CO2cooler,'H',h3_reBrayton_CO2cooler,CO2cooler_gas);

s40_reBrayton_CO2cooler=s3_reBrayton_CO2cooler;

h40_reBrayton_CO2cooler=refpropm('H','P',P4_reBrayton_CO2cooler,'S',s40_reBrayton_CO2cooler,CO2cooler_gas); % Ideal adiabatic process

h4_reBrayton_CO2cooler=((h40_reBrayton_CO2cooler-h3_reBrayton_CO2cooler)/eff_reBrayton_CO2cooler_comp)+h3_reBrayton_CO2cooler; % Through isentropic efficiency calculation

T4_reBrayton_CO2cooler=refpropm('T','P',P4_reBrayton_CO2cooler,'H',h4_reBrayton_CO2cooler,CO2cooler_gas); % compressor outlet temperature, K

s6_reBrayton_CO2cooler=refpropm('S','P',P6_reBrayton_CO2cooler,'H',h6_reBrayton_CO2cooler,CO2cooler_gas);

s10_reBrayton_CO2cooler=s6_reBrayton_CO2cooler;

h10_reBrayton_CO2cooler=refpropm('H','P',P1_reBrayton_CO2cooler,'S',s10_reBrayton_CO2cooler,CO2cooler_gas); % Ideal adiabatic process

h1_reBrayton_CO2cooler=h6_reBrayton_CO2cooler-(eff_reBrayton_CO2cooler_turbine*(h6_reBrayton_CO2cooler-h10_reBrayton_CO2cooler)); % Through isentropic efficiency calculation

T1_reBrayton_CO2cooler=refpropm('T','P',P1_reBrayton_CO2cooler,'H',h1_reBrayton_CO2cooler,CO2cooler_gas); % turbine outlet temperature, K

% performance computation

eff_motor=0.95; % generator efficiency

eff_reBrayton_CO2cooler=(h2_reBrayton_CO2cooler-h1_reBrayton_CO2cooler)/(((h4_reBrayton_CO2cooler-h3_reBrayton_CO2cooler)-(h6_reBrayton_CO2cooler-h1_reBrayton_CO2cooler))/eff_motor); % cryocooler efficiency

m_reBrayton_CO2cooler=Q_cryo_atmosphere/(h2_reBrayton_CO2cooler-h1_reBrayton_CO2cooler); %mass flow rate, kg/s

power_cryocooler=Q_cryo_atmosphere/eff_reBrayton_CO2cooler; %the power consumption of cryocooler

% Radiator area calculation

Density_atmosphere=refpropm('D','T',T_atmosphere,'P',P_atmosphere,fluid1,fluid2,fluid3,ratio); %atmosphere density, kg/m3

Cp_atmosphere=refpropm('C','T',T_atmosphere,'P',P_atmosphere,fluid1,fluid2,fluid3,ratio); % Specific heat capacity of atmosphere, J/(kg K)

Thermal_Cond_atmosphere=refpropm('L','T',T_atmosphere,'P',P_atmosphere,fluid1,fluid2,fluid3,ratio); % Thermal conductivity of atmosphere, [W/(m K)]

Dynamic_Vis_atmosphere=refpropm('V','T',T_atmosphere,'P',P_atmosphere,fluid1,fluid2,fluid3,ratio); % atmospheric dynamic viscosity [Pa s]

L_fin=0.1; % fin characteristic dimensions, m

eff_dust=0; % The coverage ratio of dust，corresponding to 0% to 100% (0~5.9mg/cm2)

Em=(0.1913*eff_dust^2)-(0.6623*eff_dust)+0.8901; %Coating equivalent emissivity, D. Keith Hollingsworth, et al.

Re_atmosphere=(Density_atmosphere*atmosphere_speed*L_fin)/Dynamic_Vis_atmosphere; % The Reynolds number on radiator panel

Pr_atmosphere=(Cp_atmosphere*Dynamic_Vis_atmosphere)/Thermal_Cond_atmosphere; % The Planck number on radiator panel

Nu_atmosphere=0.664*(Pr_atmosphere^(1/3))*(Re_atmosphere^(1/2)); % The nusselt number on radiator panel

h_atmosphere=(Nu_atmosphere*Thermal_Cond_atmosphere)/L_fin; % convection coefficient

K=20; % The number of discrete nodes in the radiator

T_radiator_reBrayton_CO2cooler=linspace(T4_reBrayton_CO2cooler,T5_reBrayton_CO2cooler,K+1); % Temperatures of each node h_radiator_reBrayton_CO2cooler=zeros(1,K+1); % The enthalpy of each node

for i=1:K+1

h_radiator_reBrayton_CO2cooler(i)=refpropm('H','T',T_radiator_reBrayton_CO2cooler(i),'P',((P4_reBrayton_CO2cooler+P5_reBrayton_CO2cooler)*0.5),CO2cooler_gas);

end

A_radiator_reBrayton_CO2cooler=0; % Radiator area, m2

for i=1:K

Aver_T_radiator_reBrayton_CO2cooler=(T_radiator_reBrayton_CO2cooler(i)+T_radiator_reBrayton_CO2cooler(i+1))*0.5-10; %The average temperature of the fins

A_radiator_reBrayton_CO2cooler=A_radiator_reBrayton_CO2cooler+(m_reBrayton_CO2cooler*(h_radiator_reBrayton_CO2cooler(i)-h_radiator_reBrayton_CO2cooler(i+1)))/((Em*5.67*10^-8*(Aver_T_radiator_reBrayton_CO2cooler^4-T_atmosphere^4))+(h_atmosphere*(Aver_T_radiator_reBrayton_CO2cooler-T_atmosphere))); %Iterative radiator area

end

Q_radiator_capacity_reBrayton_CO2cooler=(m_reBrayton_CO2cooler*(h4_reBrayton_CO2cooler-h5_reBrayton_CO2cooler))/A_radiator_reBrayton_CO2cooler; The heat dissipation capacity of the radiator, W

% Calculate the mass of the cryocooler

M_reBrayton_unit_CO2cooler=3.9985*Q_cryo_atmosphere^(-0.404); %the power density of cryocooler，W/kg，Eric Daniel Hinterman, et al.

M_radiator_unit=4.3; % The specific mass of the radiator， kg/m2

M_reBrayton_CO2cooler=Q_cryo_atmosphere*M_reBrayton_unit_CO2cooler; %cryocooler mass, kg

M_radiator_reBrayton_CO2cooler=M_radiator_unit*A_radiator_reBrayton_CO2cooler; % radiator mass, kg

% Calculate the mass of the cryochamber (Cold-head, shell, heater)

m0_ref_Cryochamber_coldhead=0.09; % Reference mass flow rate in cold-head, kg/h, Meier et al (2018).

m0_ref_Cryochamber_Shell=1.1; % Reference mass flow rate in cryochamber shell, kg/h.

m0_ref_Cryochamber_Heaters=1.1; % Reference mass flow rate in heater, kg

M0_ref_Cryochamber_coldhead=0.34; % Reference mass in cold-head, kg

M0_ref_Cryochamber_Shell=38; % Reference mass in cryochamber shell, kg

M0_ref_Cryochamber_Heaters=0.5; % Reference mass in cryochamber heater, kg

M_Cryochamber_Coldhead=M0_ref_Cryochamber_coldhead*((m_ISRU_CO2*3600/Num_Cryochamber)/m0_ref_Cryochamber_coldhead)^0.67; % cold-head mass, kg

M_Cryochamber_Shell=M0_ref_Cryochamber_Shell*((m_ISRU_CO2*3600/Num_Cryochamber)/m0_ref_Cryochamber_Shell)^0.67; % cryochamber shell mass, kg

M_Cryochamber_Heater=M0_ref_Cryochamber_Heaters*((m_ISRU_CO2*3600/Num_Cryochamber)/m0_ref_Cryochamber_Heaters)^0.67; % heater mass, kg

M_Cryochamber_tot=(M_Cryochamber_Shell+M_Cryochamber_Coldhead+M_Cryochamber_Heater)*Num_Cryochamber; % total cryochamber system mass, kg

% The total power consumption and mass of capture system Power_CO2_capture_tot=(Power_heater_ISRU+power_cryocooler+Power_valve+Power_blower); % The total power consumption for capture subsystem, W.

Power_CO2_capture_per=(Power_CO2_capture_tot/m_ISRU_CO2)/1000; % power consumption for per CO2 unit, J/g

Mass_cryocooler_system=M_Cryochamber_tot+M_radiator_reBrayton_CO2cooler+M_reBrayton_CO2cooler+M_valve+M_blower; % The total mass for capture subsystem, kg.

%%%%%%

%% CO2 Solid oxide electrolysis subsystem

% Basic parameter input

F=96485; % Faraday constant, C/mol

R=8.314; % Universal gas constant, J/(mol K), CO2→CO+0.5*O2

P_SOE_actc=0.8*10^5; % Cathode working pressure, Pa，reference to MOXIE

P_Oxygen=0.8*10^5; % Anode working pressure, Pa, reference to MOXIE

T_SOE=1123; % Reaction temperature, K

J_SOE=1200; % Current density， A/m2, Donald Rapp, et al.

P_stardand=101325; % Standard pressure, Pa

% Predefined variable

k=2; % “1” indicates the average value, “2” indicates the export side (the high risk of coking)

ratio_CO2_cal=zeros(1,k);

ratio_CO_cal=zeros(1,k);

V_SOE_tot=zeros(1,k);

V_COtoC=zeros(1,k);

P_SOE_CO2=zeros(1,k);

P_SOE_CO=zeros(1,k);

V_nernst=zeros(1,k);

Vacta=zeros(1,k);

Vactc=zeros(1,k);

Vconc=zeros(1,k);

Vcona=zeros(1,k);

Vohm=zeros(1,k);

% gas composition in the electrode interface

ratio_CO2_cal(1)=(1+(1-1*U_SOE))/2;

ratio_CO_cal(1)=(0+(1*U_SOE))/2;

ratio_CO2_cal(2)=(1-U_SOE);

ratio_CO_cal(2)=U_SOE;

for i=1:2

% partial pressure in the electrode interface

ratio_CO2=ratio_CO2_cal(i);

ratio_CO=ratio_CO_cal(i);

P_SOE_CO2(i)=P_SOE_actc*ratio_CO2; % Cathode CO2 partial pressure, Pa

P_SOE_CO(i)=P_SOE_actc*ratio_CO; % Cathode CO partial pressure, Pa

% Nernstian potential

V0=-4.4924*10^(-4)*T_SOE+1.4629; % Reversible voltage（800℃~1400℃） Forrest E. Meyen，et al.

V_nernst(i)=V0+(((R*T_SOE)/(2*F))*(log(((P_SOE_CO(i)/P_stardand)*((P_Oxygen/P_stardand)^0.5))/(P_SOE_CO2(i)/P_stardand)))); % Nernst potential, V

% Activation overpotential Houcheng Zhang，et al.

ya=2.051*10^9;

yc=1.344*10^10;

Eacta=1.2*10^5;

Eactc=1.0*10^5;

J0a=ya*exp(-Eacta/(R*T_SOE));

J0c=yc*exp(-Eactc/(R*T_SOE));

Vacta(i)=((R*T_SOE)/F)*log((J_SOE/(2*J0a))+((((J_SOE/(2*J0a))^2)+1)^0.5));% Anode activation overpotential, V

Vactc(i)=((R*T_SOE)/F)*log((J_SOE/(2*J0c))+((((J_SOE/(2*J0c))^2)+1)^0.5));% Cathode activation overpotential, V

% Concentration overpotential, Houcheng Zhang，et al.

lc=50*10^(-6); % Cathode thickness, 50um

la=500*10^(-6); % Anode thickness, 500um

le=50*10^(-6); % Electrolyte thickness, 50um

r=0.5*10^(-6); % Average pore size, m

Mco=28;

Mco2=44;

Mco_co2=2/((1/Mco)+(1/Mco2));

Dco_kn=(2*r/3)*(((8*R*T_SOE)/(pi*Mco))^0.5);

Dco2_kn=(2*r/3)*(((8*R*T_SOE)/(pi*Mco2))^0.5);

cgm_CO_CO2=(3.69+3.941)/2;

tao=T_SOE/((91.7*195.2)^0.5);

OMG_D=(1.06036/(tao^0.1561))+(0.193/(exp(0.47625*tao)))+(1.03587/(exp(1.52996*tao)))+(1.76474/(3.89411*tao));

Dco_co2=(0.0026*(T_SOE^1.5))/(P_SOE_actc*(Mco_co2^0.5)*(cgm_CO_CO2^2)*OMG_D);

Deff_CO=1/((5/0.4)*((1/Dco_kn)+(1/Dco_co2)));

Deff_CO2=1/((5/0.4)*((1/Dco2_kn)+(1/Dco_co2)));

Vconc(i)=((R*T_SOE)/(2*F))*log((1+((R*T_SOE*J_SOE*lc)/(2*F*Deff_CO*P_SOE_CO(i))))/(1-((R*T_SOE*J_SOE*lc)/(2*F*Deff_CO2*P_SOE_CO2(i))))); % cathode concentration overpotential

u_oxygen=refpropm('V','T',T_SOE,'P',(P_Oxygen/1000),'OXYGEN');

Bg=((r^2)*(0.4^3))/(18*5*((1-0.4)^2));

Vcona(i)=((R*T_SOE)/(4*F))*log((((P_Oxygen^2)+((J_SOE*R*T_SOE*u_oxygen*la)/(2*F*Bg)))^0.5)/P_Oxygen); % Anode concentration overpotential

% ohmic overpotential, Houcheng Zhang，et al.

Rohm=(la/(8.4*10^3))+(lc/(8*10^4))+(le/((3.34*10^4)*exp((-1.03*10^4)/T_SOE))); % ohmic resistance

Vohm(i)=J_SOE*Rohm; % ohmic overpotential, V

% Calculate the operating voltage

V_SOE_tot(i)=V_nernst(i)+Vactc(i)+Vacta(i)+Vconc(i)+Vcona(i)+Vohm(i);

% Nernst potential of the coking reaction: 2CO→O2+2C, Forrest Edward Meyen, et al.

V_COtoC(i)=((1.118*10^5+88.5*T_SOE)+(R*T_SOE*log(((P_Oxygen/P_stardand)^0.5)/(P_SOE_CO(i)/P_stardand))))/(2*F);

end

% Evaluate the risk of coking (Point 2)

if V_SOE_tot(2)>V_COtoC(2)

disp(' work voltage seems unreasonable');

else

disp(' The voltage point seems reasonable');

disp(V_COtoC(2)-V_SOE_tot(2));

end

% Calculate the required active area of the SOE cell.

MW_O2=0.032;

n_ISRU_O2=(mO2_require/3600)/MW_O2; % Molar flow rate of oxygen, mol/s

A_cell_tot=(n_ISRU_O2/(J_SOE/(4*F)))*10^4; %total area of the SOE cell.

% Calculate the electrolysis power, Donald Rapp，et al.

V_SOE_th=(0.5*((-0.00334*T_SOE^2)-0.57737*T_SOE+569263))/(2*F); % thermal neutral voltage, V

Power_SOE_electrolysis=V_SOE_th*J_SOE*(A_cell_tot*10^(-4)); % power consumption for SOE electrolysis, W

if P_Oxygen>P_SOE_actc % Pressure imbalance?

V_SOE_add_Compression=((R*T_SOE)/(2*F))*log(P_Oxygen/P_SOE_actc); % equivalent voltage, Eric Hinterman, et al.

Power_SOE_add_Compression=V_SOE_add_Compression*J_SOE*(A_cell_tot*10^(-4)); % Additional power consumption, Eric Hinterman, et al.

else

Power_SOE_add_Compression=0;

end

% The design of the SOE stacks

A_cell_area_signle=100; % The effective reaction area of a single cell unit is 100 cm2

N_cell_stack_single=60; % The number of effective unit batteries in a single stack

N_cell_cal=A_cell_tot/A_cell_area_signle;

N_stack_cal=A_cell_tot/(N_cell_stack_single*A_cell_area_signle);

N_stack=ceil(N_stack_cal);

N_cell=ceil(N_cell_cal);

l_numb_stack=2; % Number of electric stacks in the X direction

w_numb_stack=2; % Number of electric stacks in the Y direction

while (1<2) %internal layout (2*2，4*2，4*4，6*4 or 6*6)

if N_stack<=l_numb_stack*w_numb_stack

break

else

if l_numb_stack<=w_numb_stack

l_numb_stack=l_numb_stack+2;

else

w_numb_stack=w_numb_stack+2;

end

end

end

% SOE box size, Eric Hinterman, et al.

f_modules=1.3; % distance coefficient

w_stack_single=0.21; % Width of a single stack, m

l_stack_single=0.23; % Length of a single stack, m

t_SOE_insl=0.05; % Inner insulation material thickness, m

t_SOE_ins2=0.15; % outer insulation material thickness, m

% The three-dimensional dimensions of the inner box body, m

w_modules_inner=(f_modules*(w_numb_stack*w_stack_single))+2*t_SOE_insl;

l_modules_inner=(f_modules*(l_numb_stack*l_stack_single))+2*t_SOE_insl;

h_modules_inner=(f_modules*(0.004*N_cell_stack_single+0.05))+2*t_SOE_insl;

% (The thickness of the single-layer cell is 0.004 m, and the thickness of the cover plate is 0.05 m)

% The three-dimensional dimensions of the outer box body, m

w_modules_outer=w_modules_inner+2*t_SOE_ins2;

l_modules_outer=l_modules_inner+2*t_SOE_ins2;

h_modules_outer=h_modules_inner+2*t_SOE_ins2;

% The area of the inner box body shell, m2

A_modules_inner_base=w_modules_inner*l_modules_inner;

A_modules_inner_side=l_modules_inner*h_modules_inner;

A_modules_inner_front=w_modules_inner*h_modules_inner;

A_modules_inner_shell=2*(A_modules_inner_front+A_modules_inner_side+A_modules_inner_base);

% The area of the outer box body shell, m2

A_modules_outer_base=w_modules_outer*l_modules_outer;

A_modules_outer_side=l_modules_outer*h_modules_outer;

A_modules_outer_front=w_modules_outer*h_modules_outer;

A_modules_outer_shell=2*(A_modules_outer_front+A_modules_outer_side+A_modules_outer_base);

% The overall quality and volume of the SOE box body

V_SOE_modules_tot=w_modules_outer*l_modules_outer*h_modules_outer; % Box volume, m3

M0_cell_single=0.275; % the mass of 100cm2 cell unit, kg

M_SOE_cell_stack=(N_cell*M0_cell_single)*1.2; % Box Mass, kg

rho_efrax=230; % The density of insulation materials，kg/m3

rho_ti=4500; % The density of shell materials, kg/m3

thickness_SOE_shell=0.00635; %The thickness of shell, m

M_modules_inner=(t_SOE_insl*A_modules_inner_shell*rho_efrax)+(thickness_SOE_shell*A_modules_inner_shell*rho_ti); %The weight of the inner shell of the box, kg

M_modules_outer=(t_SOE_ins2*A_modules_outer_shell*rho_efrax)+(thickness_SOE_shell*A_modules_outer_shell*rho_ti); % %The weight of the outer shell of the box, kg

Mass_SOE_modules_tot=M_SOE_cell_stack+M_modules_outer+M_modules_inner; %The total mass of the SOE box body

% The heating loss of the box body

k_efrax_Mars=0.008; % Thermal conductivity of insulation materials

% thermal resistance

R_insl=t_SOE_insl/(k_efrax_Mars*A_modules_inner_shell);

R_ins2=t_SOE_ins2/(k_efrax_Mars*A_modules_outer_shell);

R_cond=(A_modules_outer_shell+A_modules_inner_shell)/((A_modules_outer_shell/R_ins2)+(A_modules_inner_shell/R_insl));

R_conv=1/(h_atmosphere*A_modules_outer_shell);

R_rada=0; %Ignoring thermal radiation

margin=2.5; % Redundancy coefficient

eff_SOE_heater=0.9; % heater efficiency

Power_SOE_heat_loss=(((T_SOE-T_atmosphere)/(R_cond+R_conv+R_rada))*margin)/eff_SOE_heater; %power consumption of heater

% The power consumption of the SOE subsystem

Power_SOE_system_tot=Power_SOE_electrolysis+Power_SOE_add_Compression+Power_SOE_heat_loss;

%%%%%%

%% Oxygen liquefaction and storage subsystem

T_Oxygen_Liquid_inlet=300; % Inlet temperature of the liquefier, K

P_Oxygen_Liquid_inlet=P_Oxygen; % The pressure of oxygen, Pa

% Gas pre-cooling

KK=100; % Temperature discrete nodes

T_Oxygen_precooler=linspace(T_SOE,T_Oxygen_Liquid_inlet,KK+1); % The node temperatures during the pre-cooling process

h_Oxygen_precooler=zeros(1,KK+1); % enthalpy value

for i=1:KK+1

h_Oxygen_precooler(i)=refpropm('H','T',T_Oxygen_precooler(i),'P',(P_Oxygen_Liquid_inlet/1000),'OXYGEN');

end

A_radiator_Oxygen_precooler=0;

for i=1:KK % Iterative solution for the area of the radiator

Aver_T_radiator_Oxygen_precooler=(T_Oxygen_precooler(i)+T_Oxygen_precooler(i+1))*0.5-10; %The average temperature of radiator fin.

A_radiator_Oxygen_precooler=A_radiator_Oxygen_precooler+((mO2_require/3600)*(h_Oxygen_precooler(i)-h_Oxygen_precooler(i+1)))/((Em*5.67*10^-8*(Aver_T_radiator_Oxygen_precooler^4-T_atmosphere^4))+(h_atmosphere*(Aver_T_radiator_Oxygen_precooler-T_atmosphere)));

end

M_radiator_Oxygen_precooler=A_radiator_Oxygen_precooler*M_radiator_unit;

% The mass of pre-cooler radiator, kg

% Calculate the required cooling capacity

h_Oxygen_Liquid_inlet=refpropm('H','T',T_Oxygen_Liquid_inlet,'P',(P_Oxygen_Liquid_inlet/1000),'OXYGEN');

T_Oxygen_phase_change=refpropm('T','P',(P_Oxygen_Liquid_inlet/1000),'Q',0,'OXYGEN'); % phase-transition temperature, K

delta_T_Oxygen_guoleng=3; % degree of supercooling, K h_Oxygen_Liquid_outlet=refpropm('H','T',(T_Oxygen_phase_change-delta_T_Oxygen_guoleng),'Q',0,'OXYGEN');

D_Oxygen_Liquid_outlet=refpropm('D','T',(T_Oxygen_phase_change-delta_T_Oxygen_guoleng),'Q',0,'OXYGEN');

heat_loss_Oxygen_Liquid_Tank=0.85; % Heat loss coefficient for storage tank

heat_loss_Oxygen_Liquid_pipe=0.85; % Heat loss coefficient for pipe

Q_Oxygen_Liquid_capacity=((mO2_require/3600)*(h_Oxygen_Liquid_inlet-h_Oxygen_Liquid_outlet))/(heat_loss_Oxygen_Liquid_Tank*heat_loss_Oxygen_Liquid_pipe); % Cooling capacity, W

% The parameters of the cryocooler for liquefying Oxygen

(Similar to the cryocooler for capturing CO2)

delta_T_reBrayton_O2liquid_min=5;

eff_reBrayton_O2liquid_comp=0.7;

eff_reBrayton_O2liquid_turbine=0.7;

eff_reBrayton_O2liquid_R=0.97;

delta_P_reBrayton_O2liquid_C=0.02;

delta_P_reBrayton_O2liquid_R=0.02;

delta_P_reBrayton_O2liquid_rad=0.02;

PR_reBrayton_O2liquid=2.5;

% cooling process

O2liquid_gas='NEON';

P1_reBrayton_O2liquid=400;

T2_reBrayton_O2liquid=(T_Oxygen_phase_change-delta_T_Oxygen_guoleng)-delta_T_reBrayton_O2liquid_min;

P2_reBrayton_O2liquid=P1_reBrayton_O2liquid*(1-delta_P_reBrayton_O2liquid_C);

h2_reBrayton_O2liquid=refpropm('H','T',T2_reBrayton_O2liquid,'P',P2_reBrayton_O2liquid,O2liquid_gas);

% Pressure parameters

P3_reBrayton_O2liquid=P2_reBrayton_O2liquid*(1-delta_P_reBrayton_O2liquid_R);

P4_reBrayton_O2liquid=P3_reBrayton_O2liquid*PR_reBrayton_O2liquid;

P5_reBrayton_O2liquid=P4_reBrayton_O2liquid*(1-delta_P_reBrayton_O2liquid_rad);

P6_reBrayton_O2liquid=P5_reBrayton_O2liquid*(1-delta_P_reBrayton_O2liquid_R);

% % Calculation of the recuperator

T5_reBrayton_O2liquid=280;

h5_reBrayton_O2liquid=refpropm('H','T',T5_reBrayton_O2liquid,'P',P5_reBrayton_O2liquid,O2liquid_gas);

T6_reBrayton_O2liquid=T5_reBrayton_O2liquid-(eff_reBrayton_O2liquid_R*(T5_reBrayton_O2liquid-T2_reBrayton_O2liquid));

h6_reBrayton_O2liquid=refpropm('H','T',T6_reBrayton_O2liquid,'P',P6_reBrayton_O2liquid,O2liquid_gas);

h3_reBrayton_O2liquid=(h5_reBrayton_O2liquid-h6_reBrayton_O2liquid)+h2_reBrayton_O2liquid;

T3_reBrayton_O2liquid=refpropm('T','P',P3_reBrayton_O2liquid,'H',h3_reBrayton_O2liquid,O2liquid_gas);

% compression and expansion process s3_reBrayton_O2liquid=refpropm('S','P',P3_reBrayton_O2liquid,'H',h3_reBrayton_O2liquid,O2liquid_gas);

s40_reBrayton_O2liquid=s3_reBrayton_O2liquid;

h40_reBrayton_O2liquid=refpropm('H','P',P4_reBrayton_O2liquid,'S',s40_reBrayton_O2liquid,O2liquid_gas);

h4_reBrayton_O2liquid=((h40_reBrayton_O2liquid-h3_reBrayton_O2liquid)/eff_reBrayton_O2liquid_comp)+h3_reBrayton_O2liquid;

T4_reBrayton_O2liquid=refpropm('T','P',P4_reBrayton_O2liquid,'H',h4_reBrayton_O2liquid,O2liquid_gas);

s6_reBrayton_O2liquid=refpropm('S','P',P6_reBrayton_O2liquid,'H',h6_reBrayton_O2liquid,O2liquid_gas);

s10_reBrayton_O2liquid=s6_reBrayton_O2liquid;

h10_reBrayton_O2liquid=refpropm('H','P',P1_reBrayton_O2liquid,'S',s10_reBrayton_O2liquid,O2liquid_gas);

h1_reBrayton_O2liquid=h6_reBrayton_O2liquid-(eff_reBrayton_O2liquid_turbine*(h6_reBrayton_O2liquid-h10_reBrayton_O2liquid));

T1_reBrayton_O2liquid=refpropm('T','P',P1_reBrayton_O2liquid,'H',h1_reBrayton_O2liquid,O2liquid_gas);

% The Efficiency of cryocooler for liquefying Oxygen

eff_motor=0.95; % Motor efficiency

eff_reBrayton_O2liquid=(h2_reBrayton_O2liquid-h1_reBrayton_O2liquid)/(((h4_reBrayton_O2liquid-h3_reBrayton_O2liquid)-(h6_reBrayton_O2liquid-h1_reBrayton_O2liquid))/eff_motor); % cooling efficiency

m_reBrayton_O2liquid=Q_Oxygen_Liquid_capacity/(h2_reBrayton_O2liquid-h1_reBrayton_O2liquid); % the mass flow rate of working fluid, kg/s

Power_reBrayton_O2liquid=Q_Oxygen_Liquid_capacity/eff_reBrayton_O2liquid;% the power consumption of cryocooler, W

% Heat dissipation process calculation

K=20; % The number of discrete nodes in the radiator

T_radiator_reBrayton_O2liquid=linspace(T4_reBrayton_O2liquid,T5_reBrayton_O2liquid,K+1);

h_radiator_reBrayton_O2liquid=zeros(1,K+1);

for i=1:K+1

h_radiator_reBrayton_O2liquid(i)=refpropm('H','T',T_radiator_reBrayton_O2liquid(i),'P',((P4_reBrayton_O2liquid+P5_reBrayton_O2liquid)*0.5),O2liquid_gas);

end

A_radiator_reBrayton_O2liquid=0;

for i=1:K %Iterative solution for the area of the radiator

Aver_T_radiator_reBrayton_O2liquid=(T_radiator_reBrayton_O2liquid(i)+T_radiator_reBrayton_O2liquid(i+1))*0.5-10;

A_radiator_reBrayton_O2liquid=A_radiator_reBrayton_O2liquid+(m_reBrayton_O2liquid*(h_radiator_reBrayton_O2liquid(i)-h_radiator_reBrayton_O2liquid(i+1)))/((Em*5.67*10^-8*(Aver_T_radiator_reBrayton_O2liquid^4-T_atmosphere^4))+(h_atmosphere*(Aver_T_radiator_reBrayton_O2liquid-T_atmosphere)));

end

% Calculation of the weight of the cryocooler

M_reBrayton_unit_O2liquid=3.9985*Q_Oxygen_Liquid_capacity^(-0.404); % power density of cryocooler, Kg/W

M_radiator_unit=4.3; % power density of radiator, kg/m2

M_reBrayton_O2liquid=Q_Oxygen_Liquid_capacity*M_reBrayton_unit_O2liquid;

% The mass of cryocooler, kg

M_radiator_reBrayton_O2liquid=M_radiator_unit*A_radiator_reBrayton_O2liquid; % The mass of radiator，kg

% Storage system weight calculation (Based on the results of the NASA report, calculated proportionally, Daniel M. Hauser, et al.)

% Basic weight information, kg

M0_Oxygen_storage_Tank=450; % oxygen storage tank

M0_Oxygen_storage_Tank_insulation=19; % Tank insulation materials

M0_Oxygen_storage_Jacket=115; % Vacuum jacket

M0_Oxygen_storage_support_structure=225; % Supporting structure

M0_Oxygen_storage_plumbing_insulation=27; % Pipe insulation material

M0_Oxygen_storage_Pump_valves=22; % Pump and valve

M_Oxygen_storage=(Polsgrove_Crew/23000)*(M0_Oxygen_storage_Tank+M0_Oxygen_storage_Tank_insulation+M0_Oxygen_storage_Jacket+M0_Oxygen_storage_support_structure+M0_Oxygen_storage_plumbing_insulation+M0_Oxygen_storage_Pump_valves); % The total mass of the storage tank system, kg

% The power consumption and mass of Oxygen liquefaction subsystem

Mass_O2liquid_storage_tot=M_radiator_Oxygen_precooler+M_reBrayton_O2liquid+M_radiator_reBrayton_O2liquid+M_Oxygen_storage; % subsystem total mass, kg

Power_O2liquid_storage_tot=Power_reBrayton_O2liquid; % subsystem total power consumption, W

%%%%%%

%% Martian air heat-to-electricity conversion subsystem

% basic parameter

T1_power=973; % turbine inlet temperature，K

P1_power=1000; % turbine inlet pressure，kPa

eff_power_alter=0.95; % Motor efficiency

thermal_C_316L=15; %Thermal conductivity of 316 stainless steel

PR_power=2.2;% pressure ratio in turbine

T4_power=360; % cooler outlet temperature, K

fluid_power='CO2'; % working fluid

eff_power_tur=0.86; % Turbine isentropic efficiency，Robert L. Fuller，et al (NASA)

eff_power_comp=0.84;% compressor isentropic efficiency，Robert L. Fuller，et al (NASA)

% Expansion process calculation

P2_power=P1_power/PR_power; %turbine outlet pressure, kPa

[h1_power,s1_power,D1_power,Dy1_power]=refpropm('HSDV','T',T1_power,'P',P1_power,fluid_power);

s20_power=s1_power;

h20_power=refpropm('H','P',P2_power,'S',s20_power,fluid_power);

h2_power=h1_power-(eff_power_tur*(h1_power-h20_power));

[T2_power,s2_power,D2_power,Dy2_power]=refpropm('TSDV','P',P2_power,'H',h2_power,fluid_power);

% SOEC Temperature and Pressure Regulator Calculation

h_SOE_valve=h2_power; % The throttling element is an isenthalpic process.

T_SOE_valve=refpropm('T','P',P_SOE_actc,'H',h_SOE_valve,fluid_power);

if T_SOE_valve>T_SOE

disp('The temperature at the turbine outlet is appropriate');

Power_SOE_inlet_preheat=0; % No need for preheating

else

disp('The turbine outlet temperature is lower than theSOE temperature')

h_SOE_actc=refpropm('H','T',T_SOE,'P',P_SOE_actc,fluid_power);

Power_SOE_inlet_preheat=(m_ISRU_CO2*(h_SOE_actc-h_SOE_valve))/eff_SOE_heater; % Preheating power calculation

% Match electrical power (Here, we will take ISRU oxygen production as an example)

Power_ISRU_tot=Power_CO2_capture_tot+Power_SOE_system_tot+Power_O2liquid_storage_tot+Power_SOE_inlet_preheat; % Theoretical power consumption of the entire integrated ISRU system， W

% Assuming the pressure loss in the heat exchanger

delta_P_power_R=25; % Recuperator, kPa

delta_P_power_C=10; % Cooler, kPa

delta_P_power_H=10; % Reactor, kPa

P3_power=P2_power-delta_P_power_R;

P4_power=P3_power-delta_P_power_C;

P6_power=P1_power+delta_P_power_H;

P5_power=P6_power+delta_P_power_C;

% Compression process calculation

[h4_power,s4_power,D4_power,Dy4_power]=refpropm('HSDV','T',T4_power,'P',P4_power,fluid_power);

s50_power=s4_power;

h50_power=refpropm('H','P',P5_power,'S',s50_power,fluid_power);

h5_power=((h50_power-h4_power)/eff_power_comp)+h4_power;

[T5_power,s5_power,D5_power,Dy5_power]=refpropm('TSDV','P',P5_power,'H',h5_power,fluid_power);

% Calculation of working medium mass flow rate

eff_electrical=0.9; % Power distribution redundancy 10%

m_fluid_power=(Power_ISRU_tot/eff_electrical)/(((h1_power-h2_power)-(h5_power-h4_power))*eff_power_alter); % mass flow rate, kg/s

% Calculation of the recuperator

eff_heat_recovery_R=0.83; % Design heat recovery rate，Yuzhuo Yang et al.

T3_power=T2_power-(eff_heat_recovery_R*(T2_power-T5_power)); % hot-side outlet temperature, K

[h3_power,s3_power,D3_power,Dy3_power]=refpropm('HSDV','T',T3_power,'P',P3_power,fluid_power);

h6_power=(h2_power-h3_power)+h5_power; % energy balance

[T6_power,s6_power,D6_power,Dy6_power]=refpropm('TSDV','P',P6_power,'H',h6_power,fluid_power); % cold-side outlet status

N=10; % Segmented calculation of heat transfer in the recuperator

% Thermal physical variables

T_R_high=linspace(T2_power,T3_power,N+1); % hot-side temperature, K

T_R_low=zeros(1,N+1); % cold-side temperature, K

H_R_high=zeros(1,N+1); % enthalpy value

H_R_low=zeros(1,N+1);

Dynamic_vis_R_high=zeros(1,N+1); % dynamic viscosity

Dynamic_vis_R_low=zeros(1,N+1);

Pr_R_high=zeros(1,N+1); % Planck constant

Pr_R_low=zeros(1,N+1);

thermal_R_high=zeros(1,N+1); % Thermal conductivity

thermal_R_low=zeros(1,N+1);

Density_R_high=zeros(1,N+1); % Density

Density_R_low=zeros(1,N+1);

% data importing

for i=1:N+1

[H_R_high(i),Dynamic_vis_R_high(i),Pr_R_high(i),thermal_R_high(i),Density_R_high(i)]=refpropm('HV^LD','T',T_R_high(i),'P',((P2_power+P3_power)*0.5),fluid_power);

end

H_R_low(1)=h6_power;

T_R_low(1)=T6_power;

[Dynamic_vis_R_low(1),Pr_R_low(1),thermal_R_low(1),Density_R_low(1)]=refpropm('V^LD','T',T6_power,'P',P6_power,fluid_power);

for i=1:N

H_R_low(i+1)=H_R_low(i)-(H_R_high(i)-H_R_high(i+1));

[T_R_low(i+1),Dynamic_vis_R_low(i+1),Pr_R_low(i+1),thermal_R_low(i+1),Density_R_low(i+1)]=refpropm('TV^LD','P',((P6_power+P5_power)*0.5),'H',H_R_low(i+1),fluid_power);

end

% Initial design of the cross-sectional dimensions

A_R=80; % Number of flow channels

B_R=80; % The number of the plate unit in the thickness direction

d_hyd=(4*pi*0.002^2)/(8*(0.5*pi*0.002+0.002)); % hydraulic diameter

while (1<2)

A_section_R=A_R*B_R*0.5*(pi*0.001^2); % cross-sectional area, m2

m_V_fluid_power_R=m_fluid_power/A_section_R; % mass velocity，kg/(m2*s)

Area_recuperator=0;

% Sum up the required heat exchange areas for each heat exchange node

for i=1:N

Aver_R_T=((T_R_high(i)-T_R_low(i))-(T_R_high(i+1)-T_R_low(i+1)))/

log((T_R_high(i)-T_R_low(i))/(T_R_high(i+1)-T_R_low(i+1))); Aver_Re_R_high=(m_V_fluid_power_R*d_hyd)/(0.5*(Dynamic_vis_R_high(i)+Dynamic_vis_R_high(i+1))); Aver_Re_R_low=(m_V_fluid_power_R*d_hyd)/(0.5*(Dynamic_vis_R_low(i)+Dynamic_vis_R_low(i+1)));

Aver_Pr_R_high=0.5*(Pr_R_high(i)+Pr_R_high(i+1));

Aver_Pr_R_low=0.5*(Pr_R_low(i)+Pr_R_low(i+1));

Aver_thermal_R_high=0.5*(thermal_R_high(i)+thermal_R_high(i+1));

Aver_thermal_R_low=0.5*(thermal_R_low(i)+thermal_R_low(i+1));

Aver_fd_R_high=(1.8*log10(Aver_Re_R_high)-1.5)^(-2);

Aver_fd_R_low=(1.8*log10(Aver_Re_R_low)-1.5)^(-2);

Aver_Nu_R_high=((Aver_fd_R_high/8)*(Aver_Re_R_high-1000)*Aver_Pr_R_high)/((12.7*((Aver_fd_R_high/8)^0.5)*(Aver_Pr_R_high^(2/3)-1))+1);

Aver_Nu_R_low=((Aver_fd_R_low/8)*( Aver_Re_R_low-1000)*Aver_Pr_R_low)/((12.7*((Aver_fd_R_low/8)^0.5)*(Aver_Pr_R_low^(2/3)-1))+1);

Aver_h_R_high=(Aver_Nu_R_high*Aver_thermal_R_high)/d_hyd;

Aver_h_R_low=(Aver_Nu_R_low*Aver_thermal_R_low)/d_hyd;

Aver_U_R=((1/Aver_h_R_high)+(1/Aver_h_R_low)+((0.575*0.002)/thermal_C_316L))^(-1); % Average total heat transfer coefficient

Area_recuperator= Area_recuperator+((m_fluid_power*(H_R_high(i)-H_R_high(i+1)))/(Aver_U_R*Aver_R_T)); % Total heat exchange area

end

% Calculate the length and weight of the recuperator.

Length_recuperator_core=Area_recuperator/((pi*0.5*0.002+0.002)*A_R*B_R);% The length of the core body, m

M_recuperator_core=(0.003*0.0024-pi*0.001^2)*A_R*B_R*

Length_recuperator_core*7980; % the mass of core body, kg

Width_recuperator_core=A_R*0.003;

Height_recuperator_core=B_R*0.0024;

Thickness_recuperator_case=0.01;

Thickness_recuperator_efrax=0.15; M_recuperator_case=2*7980*Thickness_recuperator_case*((Width_recuperator_core*Height_recuperator_core)+(Width_recuperator_core*Length_recuperator_core)+(Height_recuperator_core*Length_recuperator_core));% The mass of the shell body, kg M_recuperator_efrax=2*rho_efrax*Thickness_recuperator_efrax*((Width_recuperator_core*Height_recuperator_core)+(Width_recuperator_core*Length_recuperator_core)+(Height_recuperator_core*Length_recuperator_core)); % The mass of the insulation material, kg M_recuperator_tot=M_recuperator_core+M_recuperator_case+M_recuperator_efrax;% The total weight of the regenerator

% Check the pressure loss, The maximum pressure drop occurs on the high-temperature side

delta_P_high_R_cal=0;

for i=1:N

Aver_Density_R_high=0.5*(Density_R_high(i)+Density_R_high(i+1));

Aver_Velocity_R_high=(m_fluid_power/(A_R*B_R))/(0.5*pi*0.001^2*Aver_Density_R_high);

Aver_Re_R_high=(m_V_fluid_power_R*d_hyd)/(0.5*(Dynamic_vis_R_high(i)+Dynamic_vis_R_high(i+1)));

Aver_fd_R_high=(1.8*log10(Aver_Re_R_high)-1.5)^(-2); delta_P_high_R_cal=delta_P_high_R_cal+(Aver_fd_R_high)*((Length_recu

perator_core/N)/d_hyd)*Aver_Density_R_high*(0.5*(Aver_Velocity_R_high^2));

end

% Check whether the maximum pressure drop is within the required design range

if (delta_P_high_R_cal/1000)>delta_P_power_R

A_R=A_R+1; % Increase the number of flow units

B_R=B_R+1;

else

break

end

end

% Evaluation of the weight of nuclear reactors and turbomachinery

a_BRU=-5.983*((T1_power/1000)^2)+5.829*(T1_power/1000)+12.19;

M_BRU=1.8*a_BRU*(((Power_ISRU_tot/eff_electrical)/1000)^0.7)*((1+0.52*log(PR_power))/1.93); % the mass of Brayton rotating unit, kg, Tong Lu, et al.

Q_capacity_reactor=m_fluid_power*(h1_power-h6_power); % Theoretical thermal power of the reactor, W

M_reactor=0.2098*(Q_capacity_reactor/1000)+522.6; % Numerical fitting of the results from the Sandia RSMASS-D-Models, kg, Albert C. Marshall, et al.

M_sheld=0.4363*(Q_capacity_reactor/1000)+358.1-5.743*10^(-5)*((Q_capacity_reactor/1000)^2); % Numerical fitting of the results from the Sandia RSMASS-D-Models, kg, Albert C. Marshall, et al.

% Calculation of the cooling system

% The physical properties definition of the NaK coolant

Cp_NaK=904;

Dynamic_NaK=@(x)exp(0.851*(875.35-0.258*(x-273))/x)*(875.35-0.258*(x-273))^(1/3)*7.681*10^(-6);

Density_NaK=@(x)875.35-0.258*(x-273);

thermal_NaK=@(x)20.5+0.022*(x-273)-(2.05*10^(-5))*((x-273)^2);

% Calculation of the cooler (Similar to the recuperator)

eff_heat_recovery_C=0.9;

T_Nak_inlet=T3_power-((T3_power-T4_power)/eff_heat_recovery_C);

T_Nak_outlet=(eff_heat_recovery_C*(T3_power-T_Nak_inlet))+T_Nak_inlet

m_power_NaK=(m_fluid_power*(h3_power-h4_power))/(Cp_NaK*(T_Nak_outlet-T_Nak_inlet));

% Pre-set variable memory

K2=20;

T_C_high=linspace(T3_power,T4_power,K2+1);

T_C_low=zeros(1,K2+1);

H_C_high=zeros(1,K2+1);

Dynamic_vis_C_high=zeros(1,K2+1);

Dynamic_vis_C_low=zeros(1,K2+1);

Pr_C_high=zeros(1,K2+1)

Pr_C_low=zeros(1,K2+1);

thermal_C_high=zeros(1,K2+1);

thermal_C_low=zeros(1,K2+1);

Density_C_high=zeros(1,K2+1);

Density_C_low=zeros(1,K2+1);

% Import of thermophysical variables

for i=1:K2+1

[H_C_high(i),Dynamic_vis_C_high(i),Pr_C_high(i),thermal_C_high(i),Density_C_high(i)]=refpropm('HV^LD','T',T_C_high(i),'P',((P3_power+P4_power)*0.5),fluid_power);

end

T_C_low(1)=T_Nak_outlet;

for i=1:K2

T_C_low(i+1)=T_C_low(i)-((m_fluid_power*(H_C_high(i)-H_C_high(i+1)))/(Cp_NaK*m_power_NaK));

end

for i=1:K2+1

Dynamic_vis_C_low(i)=Dynamic_NaK(T_C_low(i));

thermal_C_low(i)=thermal_NaK(T_C_low(i));

Density_C_low(i)=Density_NaK(T_C_low(i));

Pr_C_low(i)=(Cp_NaK*Dynamic_vis_C_low(i))/thermal_C_low(i);

end

% Initial design of the cooling section dimensions

A_C=70;

B_C=70;

d_hyd=(4*pi*0.002^2)/(8*(0.5*pi*0.002+0.002));

while (1<2)

A_section_C=A_C*B_C*0.5*(pi*0.001^2);

m_V_fluid_power_C=m_fluid_power/A_section_C;

m_V_NaK=m_power_NaK/A_section_C;

Area_Cooler=0;

% Iterative solution for calculating the heat exchange area of the cooler

for i=1:K2

Aver_C_T=((T_C_high(i)-T_C_low(i))-(T_C_high(i+1)-T_C_low(i+1)))

/log((T_C_high(i)-T_C_low(i))/(T_C_high(i+1)-T_C_low(i+1))); Aver_Re_C_high=(m_V_fluid_power_C*d_hyd)/(0.5*(Dynamic_vis_C_high(i)

+Dynamic_vis_C_high(i+1)));

Aver_Re_C_low=(m_V_NaK*d_hyd)/(0.5*(Dynamic_vis_C_low(i)+Dynamic_vis_C_low(i+1)));

Aver_Pr_C_high=0.5*(Pr_C_high(i)+Pr_C_high(i+1));

Aver_Pr_C_low=0.5*(Pr_C_low(i)+Pr_C_low(i+1));

Aver_thermal_C_high=0.5*(thermal_C_high(i)+thermal_C_high(i+1));

Aver_thermal_C_low=0.5*(thermal_C_low(i)+thermal_C_low(i+1));

Aver_fd_C_high=(1.8*log10(Aver_Re_C_high)-1.5)^(-2);

Aver_fd_C_low=(1.8*log10(Aver_Re_C_low)-1.5)^(-2);

Aver_Nu_C_high=((Aver_fd_C_high/8)*(Aver_Re_C_high-1000)*Aver_Pr_C_high)/((12.7*((Aver_fd_C_high/8)^0.5)*(Aver_Pr_C_high^(2/3)-1))+1);

Aver_Nu_C_low=4.82+0.0185*((Aver_Pr_C_low*Aver_Re_C_low)^0.8);

Aver_h_C_high=(Aver_Nu_C_high*Aver_thermal_C_high)/d_hyd;

Aver_h_C_low=(Aver_Nu_C_low*Aver_thermal_C_low)/d_hyd;

Aver_U_C=((1/Aver_h_C_high)+(1/Aver_h_C_low)+((0.575*0.002)/thermal_

C_316L))^(-1); % Average total heat transfer coefficient

Area_Cooler= Area_Cooler+((m_fluid_power*(H_C_high(i)-H_C_high(i+1)))/(Aver_U_C*Aver_C_T)); % Total heat exchange area, m2

end

% Calculate the length and weight of the cooler

Length_Cooler_core=Area_Cooler/((pi*0.5*0.002+0.002)*A_C*B_C);

M_Cooler_core=(0.003*0.0024-pi*0.001^2)*A_C*B_C*

Length_Cooler_core*7980; % the mass of cooler core, kg

Width_Cooler_core=A_C*0.003;

Height_Cooler_core=B_C*0.0024;

Thickness_Cooler_case=0.01;

Thickness_Cooler_efrax=0.15;

M_Cooler_case=2*7980*Thickness_Cooler_case*((Width_Cooler_core*Height_Cooler_core)+(Width_Cooler_core*Length_Cooler_core)+(Height_Cooler_core*Length_Cooler_core)); % The mass the cooler shell, kg

M_Cooler_efrax=2*rho_efrax*Thickness_Cooler_efrax*((Width_Cooler_core*Height_Cooler_core)+(Width_Cooler_core*Length_Cooler_core)+(Height_Cooler_core*Length_Cooler_core)); % The weight of the insulation material，kg

M_Cooler_tot=M_Cooler_core+M_Cooler_case+M_Cooler_efrax; % cooler mass

% Check the pressure loss, The maximum pressure drop occurs on the high-temperature side

delta_P_high_C_cal=0;

for i=1:K2

Aver_Density_C_high=0.5*(Density_C_high(i)+Density_C_high(i+1));

Aver_Velocity_C_high=(m_fluid_power/(A_C*B_C))/(0.5*pi*0.001^2*Aver_Density_C_high);

Aver_Re_C_high=(m_V_fluid_power_C*d_hyd)/(0.5*(Dynamic_vis_C_high(i)+Dynamic_vis_C_high(i+1)));

Aver_fd_C_high=(1.8*log10(Aver_Re_C_high)-1.5)^(-2); delta_P_high_C_cal=delta_P_high_C_cal+(Aver_fd_C_high)*((Length_Cool

er_core/K2)/d_hyd)*Aver_Density_C_high*(0.5*(Aver_Velocity_C_high^2));

end

if (delta_P_high_C_cal/1000)>delta_P_power_C

A_C=A_C+1; %Increase the cross-sectional dimensions

B_C=B_C+1;

else

break

end

end

% Radiator area assessment

A_radiator_power=0; % The required area of the radiator, m2

for i=1:K2

Aver_T_radiator_power=(T_C_low(i)+T_C_low(i+1))*0.5-10;

A_radiator_power=A_radiator_power+(m_power_NaK*Cp_NaK*(T_C_low(i)-T_C_low(i+1)))/((Em*5.67*10^-8*(Aver_T_radiator_power^4-T_atmosphere^4))+(h_atmosphere*(Aver_T_radiator_power-T_atmosphere))); %the area of radiator, m2

end

M_radiator_tot=A_radiator_power*M_radiator_unit; % radiator mass, kg

% Calculation of the pipeline section

d_pipe_outside=5; % Initial estimate of the outer diameter, cm

while (1<2)

Thickness_pipe=0.1*d_pipe_outside; % The thickness of the pipe

d_pipe_inside=d_pipe_outside-2*Thickness_pipe; % inner diameter, cm

A_pipe_inside=0.25*pi*(d_pipe_inside/100)^2; % cross-sectional area, m2

m_V_fluid_power_pipe=m_fluid_power/A_pipe_inside; %mass velocity, kg/(m2*s)

% pressure loss calculation (from reactor outlet to turbine inlet)

L_pipe1=1.5; % Pipeline length, m

Re_pipe1=(m_V_fluid_power_pipe*(d_pipe_inside/100))/(Dy1_power);% average Reynolds number

a_pipe1=1.5/(log(Re_pipe1)); %Intermediate variable

fai_pipe1=(4.482*(1.732+5*a_pipe1))/((2^a_pipe1)*a_pipe1*(a_pipe1+1)*(a_pipe1+2)); %Intermediate variable

fd_pipe1=2/(fai_pipe1^(2/(a_pipe1+1)));% friction factor

V_pipe1=(m_fluid_power/D1_power)/A_pipe_inside; % Average speed, m/s

delta_P_power_pipe1_cal=4*fd_pipe1*(L_pipe1/(d_pipe_inside/100))*D1_power*0.5*(V_pipe1^2); % pressure loss, Pa

% pressure loss calculation (from turbine outlet to recuperator inlet)

L_pipe2=1.5;

Re_pipe2=(m_V_fluid_power_pipe*(d_pipe_inside/100))/(Dy2_power);

a_pipe2=1.5/(log(Re_pipe2));

fai_pipe2=(4.482*(1.732+5*a_pipe2))/((2^a_pipe2)*a_pipe2*(a_pipe2+1)*(a_pipe2+2));

fd_pipe2=2/(fai_pipe2^(2/(a_pipe2+1)));

V_pipe2=(m_fluid_power/D2_power)/A_pipe_inside;

delta_P_power_pipe2_cal=4*fd_pipe2*(L_pipe2/(d_pipe_inside/100))*D2_power*0.5*(V_pipe2^2);

% pressure loss calculation (from recuperator outlet to cooler inlet)

L_pipe3=1.5;

Re_pipe3=(m_V_fluid_power_pipe*(d_pipe_inside/100))/(Dy3_power);

a_pipe3=1.5/(log(Re_pipe3));

fai_pipe3=(4.482*(1.732+5*a_pipe3))/((2^a_pipe3)*a_pipe3*(a_pipe3+1)*(a_pipe3+2));

fd_pipe3=2/(fai_pipe3^(2/(a_pipe3+1)));

V_pipe3=(m_fluid_power/D3_power)/A_pipe_inside;

delta_P_power_pipe3_cal=4*fd_pipe3*(L_pipe3/(d_pipe_inside/100))*D3_power*0.5*(V_pipe3^2);

% pressure loss calculation (from cooler outlet to compressor inlet)

L_pipe4=1.5;

Re_pipe4=(m_V_fluid_power_pipe*(d_pipe_inside/100))/(Dy4_power);

a_pipe4=1.5/(log(Re_pipe4));

fai_pipe4=(4.482*(1.732+5*a_pipe4))/((2^a_pipe4)*a_pipe4*(a_pipe4+1)*(a_pipe4+2));

fd_pipe4=2/(fai_pipe4^(2/(a_pipe4+1)));

V_pipe4=(m_fluid_power/D4_power)/A_pipe_inside;

delta_P_power_pipe4_cal=4*fd_pipe4*(L_pipe4/(d_pipe_inside/100))*D4_power*0.5*(V_pipe4^2);

% pressure loss calculation (from compressor outlet to recuperator inlet)

L_pipe5=1.5;

Re_pipe5=(m_V_fluid_power_pipe*(d_pipe_inside/100))/(Dy5_power);

a_pipe5=1.5/(log(Re_pipe5));

fai_pipe5=(4.482*(1.732+5*a_pipe5))/((2^a_pipe5)*a_pipe5*(a_pipe5+1)*(a_pipe5+2));

fd_pipe5=2/(fai_pipe5^(2/(a_pipe5+1)));

V_pipe5=(m_fluid_power/D5_power)/A_pipe_inside;

delta_P_power_pipe5_cal=4*fd_pipe5*(L_pipe5/(d_pipe_inside/100))*D5_power*0.5*(V_pipe5^2);

% pressure loss calculation (from recuperator outlet to reactor inlet)

L_pipe6=1.5;

Re_pipe6=(m_V_fluid_power_pipe*(d_pipe_inside/100))/(Dy6_power);

a_pipe6=1.5/(log(Re_pipe6));

fai_pipe6=(4.482*(1.732+5*a_pipe6))/((2^a_pipe6)*a_pipe6*(a_pipe6+1)*(a_pipe6+2));

fd_pipe6=2/(fai_pipe6^(2/(a_pipe6+1)));

V_pipe6=(m_fluid_power/D6_power)/A_pipe_inside;

delta_P_power_pipe6_cal=4*fd_pipe6*(L_pipe6/(d_pipe_inside/100))*D6_power*0.5*(V_pipe6^2);

% Check whether the maximum pressure drop is within the required design range

delta_P_power_pipe_cal=[delta_P_power_pipe1_cal,delta_P_power_pipe2_cal,delta_P_power_pipe3_cal,delta_P_power_pipe4_cal,delta_P_power_pipe5_cal,delta_P_power_pipe6_cal];

delta_P_power_pipe_max_cal=max(delta_P_power_pipe_cal); % Maximum pipeline pressure drop

delta_P_power_pipe=5; % The preset maximum pressure drop within the pipeline, kPa, The impact on the cycle efficiency can be disregarded.

if (delta_P_power_pipe_max_cal/1000)>delta_P_power_pipe

d_pipe_outside=d_pipe_outside+0.5; % increase the outer diameter

else

break

end

end

% Evaluate the thickness of the insulation layer of the pipeline

Thickness_pipe_efrax=10; % The thickness of the insulation material, cm

Q_pipe_heat_loss_max=(T1_power-T_atmosphere)/((1/(2*pi*L_pipe2*k_efrax_Mars))*log(((d_pipe_outside+2*Thickness_pipe_efrax)*0.5/100)/(0.5*d_pipe_outside/100))); % (A 10 cm thick insulation layer is sufficient to ignore the heating loss in pipeline.)

% Evaluate the weight of the pipeline/valve body and insulation materials

A_pipe_efrax=(0.25*pi*(((d_pipe_outside+2*Thickness_pipe_efrax)/100)^2))-(0.25*pi*((d_pipe_outside/100)^2)); %The cross-sectional area of the insulation material, m2

A_pipe_connect=(0.25*pi*((d_pipe_outside/100)^2))-(0.25*pi*((d_pipe_inside/100)^2)); % The cross-sectional area of the connecting metal, m2

M_pipe_efrax=rho_efrax*(L_pipe1+L_pipe2+L_pipe3+L_pipe4+L_pipe5+L_pipe6)*A_pipe_efrax; % the mass of insulation material, kg

M_pipe_connect=7980*(L_pipe1+L_pipe2+L_pipe3+L_pipe4+L_pipe5+L_pipe6)*A_pipe_connect; % the mass of connecting metal, kg

N_pipe_valve=10; % The number of valves in the power system

M_pipe_valve=N_pipe_valve*M0_valve_single; % Valve mass, kg

M_pipe_tot=M_pipe_connect+M_pipe_efrax+M_pipe_valve; % pipeline system mass

% Performance and mass of the heat-to-electricity conversion subsystem

power_output=m_fluid_power*((h1_power-h2_power)-(h5_power-h4_power))*eff_power_alter; % Net output power, W

eff_power_system=(m_fluid_power*((h1_power-h2_power)-(h5_power-h4_power))*eff_power_alter)/(Q_capacity_reactor); % thermal effciciency

Mass_power_tot=(M_reactor+M_BRU+M_Cooler_tot+M_recuperator_tot+M_radiator_tot+M_sheld+M_pipe_tot); % power subsystem mass, kg

power_density=power_output/Mass_power_tot; % power density, W/kg

%% Calculate the mass of the integrated ISRU plant

Mass_integrated_ISRU_tot=Mass_cryocooler_system+Mass_SOE_modules_tot+Mass_O2liquid_storage_tot+Mass_power_tot; % integrated ISRU plant mass, kg
